# Supplementary material for: GC bias affects genomic and metagenomic reconstructions, underrepresenting GC-poor organisms
Source: Gigascience. 2020 Feb 13;9(2):giaa008. doi: 10.1093/gigascience/giaa008 (PMC7016772; doi:10.1093/gigascience/giaa008)
Supplement: giaa008_GIGA-D-19-00255_Revision_2 [file giaa008_giga-d-19-00255_revision_2.pdf]

GigaScience

Substantial GC-bias impacts genomic and metagenomic reconstructions, significantly underrepresenting GC-poor organisms

--Manuscript Draft--

**Manuscript Number:**	GIGA-D-19-00255R2
**Full Title:**	Substantial GC-bias impacts genomic and metagenomic reconstructions, significantly underrepresenting GC-poor organisms
**Article Type:**	Research
**Funding Information:**	Villum Fonden — Prof. Lars Hestbjerg Hansen<br>Aarhus Universitets Forskningsfond — Dr. Tue Kjærgaard Nielsen<br>Højteknologifonden (080-2012-3-Food genomics) — Prof. Thomas Marcus Pius Gilbert<br>Innovation Foundation Denmark (7076-00129B) — Prof. Lars Hestbjerg Hansen
**Abstract:**	Background<br><br>Metagenomic sequencing is a well-established tool in the modern biosciences. While it promises unparalleled insights into the genetic content of the biological samples studied, conclusions drawn are at risk from biases inherent to the DNA sequencing methods, including inaccurate abundance estimates as a function of genomic GC contents.<br><br>Results<br><br>We explored such GC-biases across many commonly used platforms in experiments sequencing multiple genomes (with mean GC contents ranging from 28.9% to 62.4%) and metagenomes. GC-bias profiles varied among different library preparation protocols and sequencing platforms. We found that our workflows employing MiSeq and NextSeq suffered major GC-biases, with problems becoming increasingly severe outside the 45-65% GC range, leading to a falsely low coverage in GC-rich and especially GC-poor sequences, where genomic windows with 30% GC content had over 10-fold less coverage than windows close to 50% GC content. We also showed that GC content correlates very tightly with coverage biases. The PacBio and HiSeq platforms also evidenced similar profiles of GC-biases to each other which were distinct from those seen in the MiSeq and NextSeq workflows. The Oxford Nanopore workflow was not afflicted with GC-bias.<br><br>Conclusions<br><br>These findings indicate potential sources of difficulty, arising from GC-biases, in genome sequencing which could be pre-emptively addressed with methodological optimisations provided that the GC-biases inherent to the relevant workflow are understood. Furthermore, it is recommended that a more critical approach is taken in quantitative abundance estimates in metagenomic studies. In the future, metagenomic studies should take steps to account for the effects of GC-bias before drawing conclusions, or they should employ a demonstrably unbiased workflow.
**Corresponding Author:**	Patrick Denis Browne, Ph.D<br>University of Copenhagen<br>Copenhagen, DENMARK
**Corresponding Author Secondary Information:**	
**Corresponding Author's Institution:**	University of Copenhagen
**Corresponding Author's Secondary Institution:**	
**First Author:**	Patrick Denis Browne

Powered by Editorial Manager® and ProduXion Manager® from Aries Systems Corporation

**First Author Secondary Information:**	
**Order of Authors:**	Patrick Denis Browne
	Tue Kjærgaard Nielsen
	Witold Kot
	Anni Aggerholm
	Thomas Marcus Pius Gilbert
	Lara Puetz
	Morten Rasmussen
	Athanasios Zervas
	Lars Hestbjerg Hansen
**Order of Authors Secondary Information:**	
**Response to Reviewers:**	An additional funding source was included in the Funding section.<br><br>The GigaDB dataset was cited in under 'Availability of supporting data and materials' and listed in the bibliography
**Additional Information:**	
**Question**	**Response**
Are you submitting this manuscript to a special series or article collection?	No
**Experimental design and statistics**<br><br>Full details of the experimental design and statistical methods used should be given in the Methods section, as detailed in our Minimum Standards Reporting Checklist. Information essential to interpreting the data presented should be made available in the figure legends.<br><br>Have you included all the information requested in your manuscript?	Yes
**Resources**<br><br>A description of all resources used, including antibodies, cell lines, animals and software tools, with enough information to allow them to be uniquely identified, should be included in the Methods section. Authors are strongly encouraged to cite Research Resource Identifiers (RRIDs) for antibodies, model organisms and tools, where possible.	Yes

Powered by Editorial Manager® and ProduXion Manager® from Aries Systems Corporation

Have you included the information requested as detailed in our Minimum Standards Reporting Checklist?	
**Availability of data and materials**<br><br>All datasets and code on which the conclusions of the paper rely must be either included in your submission or deposited in publicly available repositories (where available and ethically appropriate), referencing such data using a unique identifier in the references and in the "Availability of Data and Materials" section of your manuscript.<br><br>Have you have met the above requirement as detailed in our Minimum Standards Reporting Checklist?	Yes

Powered by Editorial Manager® and ProduXion Manager® from Aries Systems Corporation

Manuscript Click here to access/download;Manuscript;Main_Text.docx

Substantial GC-bias impacts genomic and metagenomic reconstructions, significantly underrepresenting GC-poor organisms

Patrick Denis Browne*
Department of Plant and Environmental Sciences, University of Copenhagen, Copenhagen, Denmark
Department of Environmental Sciences, Aarhus University, Roskilde, Denmark
pdbr@plen.ku.dk

Tue Kjærgaard Nielsen
Department of Plant and Environmental Sciences, University of Copenhagen, Copenhagen, Denmark
Department of Environmental Sciences, Aarhus University, Roskilde, Denmark
tkn@plen.ku.dk

Witold Kot
Department of Plant and Environmental Sciences, University of Copenhagen, Copenhagen, Denmark
Department of Environmental Sciences, Aarhus University, Roskilde, Denmark
wk@plen.ku.dk

Anni Aggerholm
Department of Hematology, Aarhus University Hospital, Aarhus, Denmark
anniagge@rm.dk

M. Thomas P. Gilbert
The GLOBE Institute, Faculty of Health and Biomedical Sciences, University of Copenhagen, Copenhagen, Denmark
mtpgilbert@gmail.com

Lara Puetz
The GLOBE Institute, Faculty of Health and Biomedical Sciences, University of Copenhagen, Copenhagen, Denmark
lara.c.puetz@gmail.com

Morten Rasmussen
Department of Genetics, School of Medicine, Stanford University, Stanford, CA 94305
mortenras@gmail.com

Athanasios Zervas
Department of Environmental Science, Aarhus University, Roskilde 4000, Denmark
az@envs.au.dk

Lars Hestbjerg Hansen*
Department of Plant and Environmental Sciences, University of Copenhagen, Copenhagen, Denmark
Department of Environmental Sciences, Aarhus University, Roskilde, Denmark
lhha@plen.ku.dk

*Corresponding Authors

Abstract

Background

Metagenomic sequencing is a well-established tool in the modern biosciences. While it promises unparalleled insights into the genetic content of the biological samples studied, conclusions drawn are at risk from biases inherent to the DNA sequencing

methods, including inaccurate abundance estimates as a function of genomic GC contents.

Results

We explored such GC-biases across many commonly used platforms in experiments sequencing multiple genomes (with mean GC contents ranging from 28.9% to 62.4%) and metagenomes. GC-bias profiles varied among different library preparation protocols and sequencing platforms. We found that our workflows employing MiSeq and NextSeq suffered major GC-biases, with problems becoming increasingly severe outside the 45-65% GC range, leading to a falsely low coverage in GC-rich and especially GC-poor sequences, where genomic windows with 30% GC content had over 10-fold less coverage than windows close to 50% GC content. We also showed that GC content correlates very tightly with coverage biases. The PacBio and HiSeq platforms also evidenced similar profiles of GC-biases to each other which were distinct from those seen in the MiSeq and NextSeq workflows. The Oxford Nanopore workflow was not afflicted with GC-bias.

Conclusions

These findings indicate potential sources of difficulty, arising from GC-biases, in genome sequencing which could be pre-emptively addressed with methodological optimisations provided that the GC-biases inherent to the relevant workflow are understood. Furthermore, it is recommended that a more critical approach is taken in quantitative abundance estimates in metagenomic studies. In the future, metagenomic

studies should take steps to account for the effects of GC-bias before drawing conclusions, or they should employ a demonstrably unbiased workflow.

Keywords

GC-bias, high-throughput sequencing, metagenomics, Illumina, Oxford Nanopore, PacBio

Background

Recent advances in sequencing technologies have led to the emergence of a variety of low cost per base, high-throughput sequencing (HTS) platforms [1]. Different HTS platforms vary on a number of counts, including read lengths, read quantities, biases, fidelity, cost per base and turnover time. These variations in attributes weigh in differently depending on the use case of HTS (e.g. small and large genome sequencing, genome resequencing, single-cell genome sequencing, transcriptome profiling, metagenomics studies and variant analyses [1]) and the most suitable platform, or combination of complementary platforms, is chosen.

It is well established that there are several biases in HTS data including substitution errors, insertion-deletion errors and compositional based coverage biases. For example, Illumina's MiSeq platform features substitution errors approximately 100-fold more abundantly than insertion/deletion errors, and the substitution errors occur more frequently in the first 10 nt and towards the ends of the reads [2]. Furthermore, DNA

extraction efficiency varies greatly between microorganisms, and thereby DNA extraction introduces biases into amplicon (e.g. small subunit (SSU) rRNA) surveys and metagenomics surveys [3]. However, this work focuses on coverage biases related to GC content.

Coverage biases can be introduced into HTS datasets in a variety of ways. PCR is known to be a major contributor to biases in HTS datasets [3]. It is widely known that sequencing GC-rich DNA is challenging due to its inefficient amplification by PCR [4], while GC-poor DNA can also be problematic [5, 6]. Other sample handling procedures during library preparation also contribute to coverage biases, often in a GC content dependent manner [5-9]. These biases are such that GC-rich and GC-poor sequences usually suffer from under-coverage relative to GC-optimal sequences [5, 6, 10, 11]. For instance, heat treatment (50 °C) to melt agarose gel slices prior to size selection during sample preparation can result in an under-representation of GC-poor sequences, which can be mitigated by melting agarose at room temperature [12]. Many experimental recommendations have already been made to mitigate GC-biases. Chief amongst these are recommendations aimed at reducing GC-biases introduced by PCR, such as the use of PCR-free HTS library preparation procedures when possible, choosing a less biasing PCR polymerase mixture, the use of PCR additives such as betaine to improve coverage of GC-rich regions, or trimethylammonium chloride to improve coverage of GC-poor regions and the reduction of temperature ramp rates in thermocyclers [4-8, 12, 13]. Owing to the various biasing effects of DNA processing steps, coverage evenness has been shown to vary between different HTS library preparation kits, oftentimes in a GC content related manner [5, 8]. When considering technical optimisations to mitigate

GC-bias during HTS, it is often the case that optimisations to mitigate under-coverage of high-GC regions can exacerbate the under-coverage of low-GC regions and vice versa [13].Thus it could be feasible to optimise HTS library preparation for sequencing a single microbial genome with a (approximately) known average GC content. However, this does not account for local variations in GC content within a single genome which can systematically result in very poor coverage of some loci, possibly leading to gaps in an assembly.

The focus of this work is to develop a better understanding of GC-dependent coverage biases in DNA sequencing in some of the currently most widely used HTS platforms, particularly in relation to metagenome sequencing. This is important because metagenome sequencing is being applied in a growing number of studies. Unbiased coverage in metagenome sequencing data is important since read numbers (or coverage) are used as a proxy for relative species or gene abundances in metagenomics surveys [8]. In the context of pure isolate genome (re)sequencing, unbiased coverage can be advantageous for obtaining complete coverage with relatively modest sequencing effort and many assembly algorithms do not perform optimally in the case of non-uniform coverage [14]. While it may be possible to mitigate against GC-biases with technical optimisations for single isolate genome sequencing, it will almost universally be the case that there will be a large number of DNA molecules with a wide range of average GC contents in the context of metagenome surveys. For this reason, the use of knowledge regarding the GC-bias profile of the HTS workflow employed may help to account for the effects of GC-bias during data processing. While it is generally known that GC-biases occur in HTS, it is not generally known how these

biases occur in different HTS workflows. In this work, we examine the GC-biases in five metagenome datasets and in single genome sequencing datasets of fourteen different bacteria with varying average GC contents. The implications of these biases should impact how we interpret both genomic and metagenomic data and how we design sequencing workflows in the future.

Data Description

A total of twenty shotgun genome sequencing datasets were produced using DNA isolated from fourteen different bacteria with contrasting average GC contents in order to examine the GC-dependent coverage biases inherent to five different sequencing workflows (MiSeq, NextSeq, HiSeq, Oxford Nanopore, and PacBio). Full details of which organism was sequenced according to which workflow are available in **Additional file 1**. All of these datasets have been made available in SRA under the BioProject accession number PRJNA503577. Similarly, we used five different metagenome datasets to examine GC-dependent coverage biases inherent to their workflows (Table 1), where four of these were already publicly available and one was produced as a part of another project [15], and uploaded to the SRA, under PRJNA503577, with that project's leader's consent. The library preparation protocol is an important factor when considering GC-bias in sequencing data. Therefore attention is drawn to the fact that the MiSeq and NextSeq workflows (Additional file 1) and one of the metagenome datasets (SRR8570466) were produced using very similar protocols, in contrast to the long read libraries and the other Illumina datasets (HiSeq genome

sequencing and the remaining metagenome libraries). None of the Illumina datasets were derived from PCR-free libraries while the PacBio and Nanopore data were.

We also produced digital droplet PCR (ddPCR) data using three different primer sets targeting subsections of two single copy genes and the 16S rRNA gene on the chromosome of *Fusobacterium sp*. C1. The amplicons had different GC contents and ddPCR was used to assess the copy number of the 16S rRNA gene per chromosome. Finally, we produced MiSeq reads from triplicate equimolar mixtures of two 5.3 kb PCR products amplified from *Fusobacterium sp*. C1 in order to confirm the occurrence of GC-dependent coverage biases independently of the genomic background. These MiSeq reads were also uploaded to the SRA under PRJNA503577.

Analyses

Fusobacterium sequencing exemplifies under-coverage of GC-poor loci

We chose *Fusobacterium sp*. C1 for a wide range of experiments related to GC-bias to build a complete picture of how GC-biases manifest in the sequencing of a GC-poor bacterial genome. These experiments encompassed genome sequencing using five different workflows (MiSeq, NextSeq, HiSeq, PacBio and Nanopore), MiSeq sequencing of long-range (5.3 kb) PCR amplicons and ddPCR to validate the SSU rRNA copy number.

Assembly of the *Fusobacterium sp.* C1 sequencing data resulted in one complete circular chromosome, 2,032,704 bp in length, and two probable plasmids, 1,964 and 2,272 bp in length. The probable plasmids were omitted from coverage analyses due to uncertain stoichiometric ratios with the chromosome (see Methods). Hereafter the term C1 assembly refers only to the approx. 2.0 Mb contig. The C1 assembly had a relatively low GC content at 28.9%. Unsupervised annotation indicated that there were 1856 CDSs, 66 tRNA genes and 28 rRNA genes in 9 rRNA loci.

Coverage of the C1 assembly by all five sequencing workflows is illustrated in **Fig. 1**. In the MiSeq, NextSeq, HiSeq and PacBio workflows, it is apparent that there are numerous coverage spikes, especially in the vicinity of rRNA loci. These coverage spikes appear to be much sharper in the MiSeq and NextSeq datasets than in the HiSeq and the PacBio datasets, with the biggest coverage spikes in the MiSeq and NextSeq data co-occurring very closely with changes in GC content in rRNA loci. For the GC-biased workflows (MiSeq, NextSeq, HiSeq and PacBio), the coverage depths at the rRNA loci vary between 5.1- and 8.0-fold higher than background coverage depths (MiSeq – 8.0; NextSeq - 5.1; HiSeq - 6.2 PacBio – 8.0), while for the Nanopore dataset, this ratio was 1.0 (calculations are detailed in https://github.com/padbr/gcbias). In contrast to the other four workflows, the Nanopore dataset had comparatively even coverage apart from one broad coverage spike near the end of the linear representation of the chromosome (**Fig. 1**). The broad coverage spike in the Nanopore workflow had seemingly no relationship to local GC content.

To verify the coverage spikes and to rule out the possibility of misassembly resulting in an underestimation of the number of rRNA loci, further experiments were performed.

Firstly, ddPCR was used to compare the ratio of a region of the small SSU rRNA to two other single copy genes. Ratios of 9.4 and 11.0 SSU rRNA were found to the two other loci, respectively, by ddPCR. These ratios (9.4 and 11.0) are close to the number of rRNA loci annotated in the C1 assembly. This supports the inference that there are about nine rRNA loci in the C1 chromosome as presented in the assembly, and dispels the notion that there are significantly more than nine (up to 72 based on 8.0-fold over-coverage) rRNA loci based on the abovementioned high relative coverage of the rRNA loci in four out of the five sequencing datasets.

Secondly, the MiSeq workflow was used to sequence an equimolar mixture of two 5.3 kb PCR products of two loci from *Fusobacterium sp.* C1 with GC contents of 30.2% (a locus containing coding-sequences and intergenic sequences) and 45.5% (a locus containing rRNA-encoding genes and intergenic regions). This approach was to facilitate separating local GC content from global genome signatures, such as the fact that the majority of the genome is GC-poor, while primarily only the rRNA loci are GC-optimal. The 45.5% GC fragment evidenced higher coverage with 4.14-, 10.63- and 5.39-fold (3 replicates) more reads mapping to it than to the 30.2% GC fragment. This further supports the hypothesis that there are coverage biases related to GC content inherent in our Nextera XT/ MiSeq workflow. Further information on this experiment, and a plot illustrating sequencing coverage overlaid upon GC content are available in **Additional files 2 - 4**.

Manifestation of GC-biases in various HTS workflows

We then examined GC-related coverage biases in the MiSeq-based genome sequencing of ten different bacteria with average GC contents ranging from 28.9% to 62.4% (**Additional file 1**). These were all produced using the same workflow involving transposon-mediated cleaving and tagging (tagmentation) of DNA and 14 PCR cycles. Coverage was assessed in 500 bp wide sliding windows, and the coverage was normalised by dividing by the average coverage of the 49% GC genomic windows. The choice of 49% was simply because all bacteria sequenced in this work have sufficient (at least 3) numbers of 500 nt genomic windows with 49% GC content. The normalised coverage was log-transformed in the plots presenting the results. In every case, sequencing libraries were prepared following the same workflow with the Nextera XT DNA library prep kit. From plots of normalised relative coverage versus GC content (**Fig. 2**), it can be seen that a local GC content of between approx. 50%-60% is optimal, and the relative coverage decreases considerably as the local GC content becomes more dissimilar from the optimal range. The relatively small error-bars (standard deviations) seen in **Fig. 2** indicate that there generally isn't considerable variation in relative coverage among the various individual 500 nt genomic windows of the same GC content, suggesting that relative coverage and local GC content are tightly correlated. This corroborates the sharper peaks of the MiSeq dataset compared with the HiSeq and PacBio datasets (**Fig. 1**). An overlaid plot (**Additional file 5 part A**) from all experiments in **Fig. 2** shows that the GC content related coverage bias is dependent primarily on the local GC content and is not affected in a big way by other factors such as global GC content or other sequence signatures. In fact, a quadratic curve could be

fitted reasonably well (R^2 = 0.97) to the overlaid plot of normalised relative coverage versus local GC content (**Additional file 5 part A**).

The median qualities (Phred scores) of MiSeq reads were high for reads with GC contents below approximately 65%, but decreased above this GC level (**Additional file 6**). This decrease in quality above 65% GC content resulted in reads with high-GC content being more affected by quality filtering than reads with moderate or low-GC content (**Additional file 7**).

We also have NextSeq datasets derived from Nextera XT libraries for the genome sequencing of five different bacteria, ranging in GC content from 28.9% to 63.0% (**Additional file 1**, **Fig. 3**). This data was produced similarly to the MiSeq data where library preparation involved tagmentation and 14 PCR cycles. In these, the normalised relative coverages decreased as the local GC contents decreased below ca. 55% in all but the *Aminobacter* dataset. *Aminobacter* had the highest global GC content (63%) in this study and its NextSeq dataset evidenced almost no coverage bias related to local GC content between 41% and 74%. The *Rhizobium* NextSeq dataset, with local GC content ranging from 39% to 70% showed decreased relative coverage as the local GC content decreased below 55%, and very little coverage bias above 55% local GC content. The five NextSeq datasets do not overlay upon each other (**Additional file 5 part B**) as well as the ten MiSeq datasets (**Additional file 5 part A**), as judged visually, nor do they align as closely with the quadratic curve of best fit (R^2 = 0.91) (**Additional file 5 part B**). The small error bars seen in the NextSeq plots (**Fig. 3**) corroborate the sharpness of the peaks in **Fig. 1**, indicating that local coverage of the NextSeq data, as was also the case for the MiSeq data, is tightly correlated with local GC content.

NextSeq reads were not affected by quality filtering with respect to GC content in the manner in which the MiSeq reads were (**Additional file 7**), despite the fact that these reads had lower quality scores where their GC contents were over c.a. 65% (**Additional file 6**).

Two PacBio datasets (produced using a PCR-free protocol), from *Fusobacterium* and *Sphingobium* which differ greatly in global GC content, were also examined for coverage biases (**Fig. 3**). The *Sphingobium* PacBio dataset showed almost no GC-bias between 38% and 76% local GC content and very consistent coverage as judged by the very small error bars in **Fig. 3**. Below 40% local GC content, the *Fusobacterium* dataset evidenced lower relative coverage, while the large error bars in this range show that the relative coverage is highly variable, indicating that factors other than local GC content have an influence on the relative coverage in the PacBio sequencing workflow in a predominantly low GC content background. A single HiSeq dataset for *Fusobacterium* also evidenced several fold- (up to almost 10 fold-) under-coverage and large error bars for windows with less than 40% local GC content (**Fig. 3**), indicating that the HiSeq workflow's relative coverage is also affected by factors other than local GC content. The HiSeq dataset evidenced normal relative coverage from 40% to 55% local GC content. This HiSeq data derived from a workflow involving sonication to shear DNA, followed by blunt-ending, adapter ligation and 11 cycles of PCR.

Two Nanopore datasets were produced with PCR-free workflows for organisms with low and high global GC contents, *Fusobacterium* (28.9% GC) and Aminobacter (63.0% GC). Both of these datasets evidenced no major relative coverage biases related to local GC content (**Fig. 3**) and the error bars were generally quite small, suggesting that

the Nanopore workflow gives very even coverage across a wide range of GC contents and in different local genomic contexts.

GC-biases in metagenome datasets

The effects of GC content were also investigated in five independent metagenome datasets. These datasets were from different environments where the microbial communities would be expected to have different complexities. Furthermore, the datasets were prepared following different workflows and using different sequencing platforms (Table 1). Given that there were no 1% wide GC-bins common to all contigs in these assemblies, the GC-biases were presented in a different manner to the single genome datasets above (see Methods), by presenting log-transformed coverage ratios in pairs of 1% wide GC-bins within each contig in 3-dimensional plots (**Additional files 8 - 12**). In these, it can be seen that the GC-biases differed considerably between datasets. In ERR526087 (human female fecal metagenome), it is seen that GC-bins of approx. 45% received optimal coverage, while the relative coverage decreased as the GC content increased above or decreased below this optimum. In SRR8570466 (moving bed biofilm reactor metagenome) there was little or no GC-bias between 40% and 70% while the relative coverage decreased outside of this range. In SRR5035895 (kelp-associated biofilm metagenome), the relative coverage increased with increasing GC content between 25% and 67%. In SRS049959 (human male fecal metagenome), optimal coverage was seen for GC contents between 17% and 36% and relative coverage decreased as the GC content increased above 36%. In the SRR7521238 (vulture gut) metagenome dataset, optimal coverage occurred between about 50% and

60% GC content, with the relative coverage decreasing as the GC content increased above or decreased below this optimal range.

Discussion

The overarching aim of this study was to improve the general understanding about the impacts that GC-related coverage biases may have on abundance estimates of species or functions / pathways in HTS-based shotgun metagenomics experiments. However, we firstly presented results describing GC-biases in the sequencing of single bacterial genomes. The reason for this is that subsets of bacterial chromosomes with differing GC contents are equally abundant, if one can assume minimal effects from replication forks, which facilitates a thorough investigation of GC-biases within a single molecule. The *Fusobacterium* sp. C1 genome sequence presented here was from an isolated representative of the dominant operational taxonomic unit in new world vulture gastrointestinal tracts detected by amplicon analysis (SSU rRNA) [16]. In our attempt at sequencing this strain's genome we found such severe coverage biases seemingly linked to GC content that we considered it pertinent to seek further validation of the copy number of rRNA loci via ddPCR. The problem of coverage of the rRNA loci in particular arose because the majority of CDSs and intergenic regions in *Fusobacterium sp.* C1 have low-GC contents, while its rRNA genes are typical with respect to other prokaryotes in having balanced (between 50% and 60%) GC contents (**Additional file 13**, [17]). This discrepancy in GC contents is almost certainly responsible for the under-coverage of the majority of the C1 assembly relative to the rRNA loci. From our results,

we would predict that SSU rRNA amplicon studies would be less sensitive to GC-bias than shotgun metagenomics owing to the narrow range in GC content typically associated with SSU rRNA (**Additional file 13**) which also corresponds to the optimal GC range in our NexteraXT/MiSeq workflow. This is not to downplay the extent of other biases in amplicon surveys, such as those related to DNA extraction from a wide variety of cell types, (degenerate) primer annealing and variations in SSU rRNA copy number between species [3, 18]. However, in a shotgun metagenome survey (which also suffers from the abovementioned DNA extraction biases) the under-coverage of the predominantly GC-poor regions of *Fusobacterium* sp. C1's genome would, based on results presented here, result in a severe underestimation of its relative abundance. It was this notion that prompted us to delve deeper into assessing the relationships between GC content and coverage in various HTS platforms.

Results presented here showed that local GC content correlated well with coverage biases in MiSeq and NextSeq datasets produced from libraries made using Nextera XT kits. Furthermore, after normalising coverage data and performing polynomial regression, approximate descriptions of GC-bias profiles in mathematical terms were derived for our MiSeq and NextSeq workflows. The quadratic equations presented in **Additional file 5** are perhaps not the most accurate descriptions of GC-bias possible, based on deviations of the data points from the quadratic curves, especially at the extremities of the explored GC content. This suggests that the GC-biasing mechanism(s) don't follow exactly the relationships implied by the quadratic expressions. Nonetheless, the proximity of the data points to the quadratic regression curves (**Additional file 5**) is quite good considering that coverage would, in theory, be

described in such plots (**Additional file 5**) as the line “y=0” if there was no coverage bias due to local GC content. It could be argued that there is a combination of at least two different GC-biasing mechanisms at work in the MiSeq workflow. One of these is linked to the fact that reads with high-GC content generally have lower quality (Phred scores) (**Additional file 6**) and quality filtering affected high-GC reads (c.a. > 65% GC) more than other reads with balanced and low GC contents (**Additional file 7**). It could be the case that the reduction in the proportions of reads passing quality filtering between around 65% to 80% GC content in the *Agrobacterium*, *Ensifer*, and *Sphingobium* MiSeq datasets could be predominantly responsible for the corresponding declines in the relative coverage seen above 65% GC content (Figure 2). The NextSeq reads did not show such a trend of quality filtering disproportionately affecting reads of between 65% and 80% GC content. This may explain why the NextSeq datasets have unchanging relative coverage between about 55% and 72% GC content, at least for the *Rhizobium* and *Aminobacter* datasets (**Figure 3**). The lower relative coverage at low-GC contents evident in the MiSeq and NextSeq datasets is not linked to quality filtering of the reads, indicating that the mechanisms biasing against GC-rich and GC-poor windows are different. It can also be concluded that quality filtering was not largely responsible for the GC-bias in the HiSeq dataset (Figure 3, Additional file 7), though our HiSeq data is representative of only low and moderate GC contents. Though it is clear that the quality filtering resulted in at least some of the under-coverage seen at higher GC contents, we still maintain that it is correct to refer to this effect as "GC-bias", as quality filtering is a necessary part of data analysis and the low quality is related to the sequencer not being capable of calling bases with high confidence in high-GC reads.

GC-related coverage biases were seen in HiSeq and PacBio workflows (at least for *Fusobacterium* sp. C1) in a manner clearly different to an approximate polynomial curve (**Fig. 3**). Another facet of the differences between GC-bias profiles among HTS workflows is seen in the error bars of the plots of the HiSeq and PacBio datasets which, for low-GC regions (< 40% GC) are large in comparison with the error bars seen in the plots of the MiSeq, NextSeq, and Nanopore datasets. Based on the sharpness of the peaks (indicating coverage) in **Fig. 1** corresponding to changes in GC content for MiSeq and NextSeq data in comparison with the wider corresponding peaks of PacBio and HiSeq coverage plots, it is possible that another factor co-governing coverage biases in the HiSeq and PacBio workflows is proximity to a region of balanced (c.a. 50% to 60%) GC content. It could possibly be the case that linkage of GC-poor loci to GC-optimal loci (c.a. 50%) results in more efficient recovery of low-GC DNA proximal to rRNA loci, if it is the case that heat production from bead-beating (partially) denatures DNA before it is bound to a silica column. This would be similar to the bias introduced against GC-poor loci during DNA extraction from agarose gel slices described elsewhere [12]. This was not investigated further here as we aimed to investigate GC-biases inherent to HTS workflows without going into details of which mechanisms within each workflow introduced biases.

The even coverage of the Nanopore datasets over a wide range of GC contents, albeit for only two organisms with very different global GC contents, is promising, especially for metagenome sequencing where long reads will greatly simplify assembly. The application of Nanopore technology to metagenomics is currently still limited by cost, read quality and throughput, though this situation has been improving considerably ever

since the development of the technology [19]. In the meantime, when a combination of sequencing platforms are being used (e.g. if using long reads to improve assembly in combination with short reads to provide high coverage), there is the possibility that Nanopore reads, or reads derived from any other demonstrably unbiased HTS workflow, could be used as an internal standard to evaluate and perhaps correct for GC-biases or other coverage biases from cheaper or more high-throughput, but biased, workflows.

The examination of the GC-biases in five different workflows is informative even for single genome sequencing. It is perhaps unsurprising that the PCR-based Nextera XT workflow producing libraries for MiSeq and NextSeq would be heavily GC-biased. It has been reported previously that extreme GC content can complicate a single genome sequencing project [6, 9, 13] and our results are illustrative of why this is the case, showing, for example, 10-fold or worse under-coverage of GC windows under 30% in MiSeq data. However, the lack of PCR in the library preparation for the PacBio workflow did not completely alleviate GC-bias, although it would appear to have been lessened, and there exists the possibility that the primary bias in this workflow could have been introduced at the stage of DNA isolation. It is, perhaps, curious that the PacBio and HiSeq workflows gave similar profiles of GC-bias despite the PacBio workflow having no PCR and the HiSeq workflow having 11 PCR cycles. It is commonly taken as best practice to use a PCR-free sequencing library preparation method for metagenomic studies when sample biomass isn't limiting [12, 20], but, nonetheless, it can be seen that PCR is not the only major contributor to GC-bias in HTS.

We have shown the occurrence of GC-biases in five independent metagenome datasets in order to illustrate the points also addressed with the single genome experiments, namely that there are GC-dependent coverage biases which manifest in a manner dependent upon the particular workflow employed. The production of these datasets encompassed a range of different sequencing technologies and library preparation workflows with between four to fourteen PCR cycles in each case. Because of this, the profile and severity of GC-biases differed considerably between these datasets (**Additional files 8 - 12**). Owing to the fact that PCR is commonly cited as a major contributor to GC-bias [13], it is often recommended to reduce the number of PCR cycles (or to eliminate PCR altogether) as far as sample biomass and other experimental constrains allow [21]. We did not design our experiments nor analyses to assess the individual contributions to GC-bias from any of the individual steps of library preparation, but work here and elsewhere also indicates that there are sources of GC-bias other than PCR [9, 21]. The analysis of the metagenome datasets reiterated the observation from the single genome sequencing datasets where GC-biases differ between different sequencing workflows and highlights how important it is to consider this before committing to an experimental workflow. Furthermore, if the GC-bias profile in a metagenome dataset is assessed following an assembly of the data, it may be possible to estimate parameters to be used to reduce abundance estimate errors due to GC-bias. However, we did not explore the application of corrections to account for GC-bias during data processing in this work.

Even for sequencing projects employing the same sequencing technology with the same library preparation workflows, it must be considered that there could be within-

and between-lab variation. For instance, it is possible that differences in equipment / instrumentation (e.g. in ramp rates of thermocyclers [13]) between labs otherwise employing the same protocols could alter the GC-biases. And naturally, the use of different HTS workflows (including the use of different library preparation kits, different fragmentation methods, different DNA polymerases etc.) would be expected to alter the relationships between GC content and coverage considerably [5-8, 12, 13]. As discussed in the introduction, PCR additives can be used to mitigate the under-coverage of low- or high-GC regions, but these approaches tend to exacerbate biases in other regions. Thus, such an approach can possibly find utility in single genome sequencing, but is not viable for metagenome sequencing. For this reason, it may be even more important in metagenomic studies to understand the GC-biases inherent in a sequencing workflow and account for them during data analysis.

The relationships between local GC content and relative coverage presented here for single bacterial genome sequencing agree, at least qualitatively, with data published elsewhere [11, 13], in that low and high-GC regions suffer from under-coverage in comparison with GC neutral regions. The strong bias against GC-poor loci, as in the genome of *Fusobacterium* here, was previously reported for the genome of the important pathogen *Plasmodium falciparum* (19.3% GC average) [5]. However, our results also contradict some other findings, such as where it was reported that 30% GC regions were more highly covered than 50% GC regions for MiSeq and PacBio data [9]. Those data sets were produced in workflows employing different library production protocols to our in-house data, illustrating the point made above, that there can be differences in coverage biases between different labs which employ different HTS

workflows, necessitating that any attempt at accounting for GC-biases must be calibrated to the protocols and equipment in each lab separately.

Nonetheless, we propose that strategies similar to the coverage normalisation procedures described herein (https://github.com/padbr/gcbias) could be a basis for generating lab-specific and protocol-specific descriptions of GC-bias, at least in qualitative terms. However, it is uncertain how consistently HTS workflows will conform to previously derived descriptions of GC-bias profiles for each individual workflow, as illustrated by the differences in the GC-biases between our NextSeq datasets. For this reason, we would recommend extreme caution in naively using polynomial / quadratic regression as a model to describe normalised local-GC content versus coverage in NexteraXT libraries sequenced with MiSeq or NextSeq despite how consistently we have shown this to describe GC-biases in such datasets from our group. One major drawback of our coverage normalisation procedures for bacterial genome sequencing GC-bias analyses is that it relies on normalising to the average coverage in a single 1% wide GC-bin (49% GC) for each molecule (chromosome). This would make it not feasible to have a single normalisation procedure that would work on genomes with very low to very high average GC contents as not all of these would have a sufficient number of 49% GC windows, and was the reason why we employed a different protocol to visually present the GC-biases in metagenome datasets. It could be possible to account for GC-biases in a metagenome dataset by characterising the biases as we have described and adjusting the relative coverage levels in a GC-dependent manner. Alternatively, a workflow inherently devoid of GC-bias, such as the Nanopore

sequencing workflow used here, could be used for metagenome sequencing, albeit at a higher cost or with lower coverage.

Potential implications

HTS is being applied ever more frequently in genome and metagenome sequencing based investigations. GC-biases are prevalent in HTS datasets produced from a wide variety of library building and sequencing platforms, with the notable exception of the Nanopore workflow used here. Some of the most obvious and serious implications of uneven coverage in HTS include skewed abundance estimates in metagenomics projects and the presence of gaps in genome assemblies due to systematic under coverage of low- or high-GC loci. To our knowledge, no metagenomics data analysis pipeline currently accounts for GC-biases for the purposes of estimating species, gene or pathway (etc.) abundances. While many researchers may be aware of the existence of GC-biases, the manifestation of GC-biases differs between HTS workflows, which may make it difficult for researchers to understand how their HTS workflows are affected by GC-bias. For instance we show less than 10-fold under-coverage for 30% GC windows, worsening to around 30-fold under-coverage for 20% GC windows in our MiSeq workflow. To address this issue, we have, along with this article, made available a bioinformatics pipeline that can facilitate researchers in easily getting an understanding, at least in qualitative terms, of the GC-biases in their HTS workflows, using data they may already have to hand.

Such understanding of GC-biases can be used to find solutions to various problems. For example, if a lab / research group routinely performs a lot of genome sequencing followed by assembly, they may supplement their normal library preparation protocol, for instance with PCR additives, to alter GC-biases, using the pipeline here to understand the effects of their alterations. This approach could facilitate making smarter choices in the lab to maximise the fitness for purpose of datasets or making workflows more cost effective. Alternatively, if feasible, they may employ an inherently less biases (unbiased even) work flow, such as the Nanopore workflow here. Another obvious implication of understanding GC-biases could be a better interpretation of metagenomic data, or possibly even correcting abundance estimates for GC-biases. In cases of HTS workflows featuring extreme GC-biases, such as seen for Nextera XT followed by MiSeq or NextSeq sequencing, it would be extremely advantageous to account for GC-biases during data analysis, while for other HTS workflows subject to very little GC-bias (e.g. the Nanopore workflow), it may prove futile to attempt to improve abundance estimate accuracies by accounting for GC-bias. A less obvious approach in the field of metagenomics would be to actually take advantage of GC-bias. For instance, it may be possible in some cases to use additives in the PCR step of metagenome library preparation to adjust the GC-bias in favour of the average GC content of a non-culturable organism for which a de novo assembly is desired from metagenome reads. Ultimately, knowledge regarding the biases inherent in the production of a dataset can yield options to optimise the suitability of the data for the research questions and facilitate a more accurate interpretation of the data during analysis.

Methods

Strain isolation

The model organism primarily and initially used to investigate coverage biases, *Fusobacterium sp.* C1, was isolated from a frozen sample of the contents of a vulture's large intestine. The sample was thawed, serially diluted and spread on anaerobic medium plates (Statens Serum Institut) in an anaerobic jar with an environment consisting of 90% N_2 and 10% H_2 at 37 ^{O}C. The isolate was purified with several rounds of streaking in the same conditions.

Genome sequencing, assembly and annotation

DNA isolation was performed using the UltraClean Microbial DNA isolation kit (MoBio) in all cases except for the ddPCR experiment and Nanopore library preparations for which high molecular weight DNA was isolated using the Genomic Mini AX Bacteria kit (A&A Biotechnology). For MiSeq (2x251 bp paired reads) and NextSeq (2x151 bp paired reads), libraries were prepared using the Nextera XT V2 Sample preparation kit (Illumina) according to the manufacturer's instructions with the modification of increasing the number of PCR cycles from 12 to 14 during the library amplification step.

In the HiSeq workflow, genomic DNA was sheared using a Bioruptor® XL (Diagenode, Inc), with 6 rounds of 15 seconds sonication separated by 90 second intervals. Sheared DNA was converted into Illumina compatible libraries using a NEBNext library kit (E6070L) using adapters described elsewhere [22]. Following this, the library was amplified with 11 cycles of PCR using AmpliTaq Gold polymerase (Applied Biosystems,

Foster City, CA) and cleaned using Agencourt AMPure XP (Beckman Coulter, Inc) bead purification, following the manufacturer's protocol.

For Nanopore and PacBio sequencing, high molecular weight (HMW) DNA was routinely extracted from liquid cultures of bacteria using the Genomic Mini AX Bacteria kit (A&A Biotechnology (060-60)). Nanopore libraries were prepared with the Rapid Sequencing kit (SQK-RAD004) and sequenced on a FLO-MIN106 flow cell. Reads were basecalled using Albacore V.2.3.0. PacBio sequencing was performed as described elsewhere [23], with sequencing libraries being prepared using a PCR free ligation of sequencing adapters to fragmented blunt-ended double-stranded DNA.

Adapter contaminants and low quality 3' ends were trimmed from the Illumina reads with Cutadapt v1.8.3 [24]. Nanopore reads were cleaned with Porechop V.0.2.3. PacBio reads were quality filtered, adapter filtered and converted from *.bax.h5 to fastq format using pls2fasta from the blasr package (v1.0.0.126414) [25]. Paired Illumina reads were merged with AdapterRemoval v2.1.0 [26] and assembled using SPAdes v3.10 [27]. For *Fusobacterium* sp. C1, assembly was performed with Unicycler v0.4.3 running SPAdes v3.11.0 and racon using only NextSeq and Nanopore reads. For *Sphingobium herbicidovorans* MH, a publically available assembly was used (CP020538-42). Where necessary, the RAST annotation server [28] was used to predict coding sequences (CDSs), rRNAs and tRNAs. Circular plots of genome assembly and annotation information were made using BRIG [29]. All genome sequencing reads generated in this work were deposited to SRA under the BioProject number PRJNA503577.

Coverage evenness assessment of isolate genome sequencing

Cleaned, quality filtered sequencing reads were aligned to their draft genome assemblies using bwa-mem v0.7.15-r1140 [30] for MiSeq, NextSeq and HiSeq reads or minimap2 [31] for Nanopore and PacBio reads. For paired reads, the merged and unmerged reads were mapped separately to their reference assemblies and the resulting alignment files were merged using samtools merge [32]. Secondary and supplementary alignments were removed using samtools view with the flag '-F 0x900'. The coverage at each nucleotide position was calculated using samtools v1.4.1 (depth -a option) [32]. Since abnormal coverage (relative to the chromosome(s)) can arise from multicopy plasmids, phages, unresolved repeats [10] etc., contigs shorter than 10 kb were discarded and then contigs (longer than 10 kb) with abnormal coverages were identified using a modified z-score based on median absolute deviation with a threshold of 10 [33] and removed from further analyses. The exceptions were that the length cutoff was increased to 100,000 for the *Aminobacter* assembly due to highly variable coverage in contigs between 10,000 bp and 100,000 bp, and the elements annotated as plasmids for *Sphingobium herbicidovorans* MH were manually removed. Local GC contents and sequencing coverages were calculated in 500 nt sliding windows, in a similar approach to elsewhere [13], unless otherwise specified. Coverages were normalised by binning the coverage windows by GC content, with bins being 1% wide, and the coverages of all windows were divided by the average coverage of the windows binned at 49% GC. The choice of 49% GC as a baseline was due to the fact that all of our in-house datasets had at least three 500 nt windows with this GC content. GC percentage windows with less than three points were discarded. Polynomial regression

was performed on the log-transformed average coverage of each 1% wide GC-bin using the polyfit function of python's numpy package with two degrees of polynomial fitting and weights set to the number of windows for each 1% wide GC-bin. The conclusions derived from the results presented here are not affected by the choice of a sliding window width of 500 nt. This was asserted by repeating the analyses using window sizes ranging from 50 nt to 5000 nt (Additional file 14). The deviations indicated by the error bars were a little larger for smaller windows, while there were fewer windows with less extreme GC contents when looking at large window sizes. Nonetheless, the overall trends in the analyses remain very consistent regardless of window size. Further information, including source code for in-house scripts, is available at https://github.com/padbr/gcbias.

Metagenome assembly and coverage evenness assessment

Metagenome datasets were retrieved from several sources. Datasets ERR526087 (2 x 100bp) and SRR5035895 (2 x 300 bp) were retrieved with the fastq-dump utility of the SRA toolkit V.2.9.0. The longest reads in these datasets were split in half in order to retrieve the original read pairs, while shorter reads, presumably trimmed for quality or removing technical sequences, were discarded since the read pairs were concatenated without annotation of the concatenation point making it impossible to recover the original paired reads. SRS049959 (2 x 100bp) was downloaded from the human metagenome project website with ftp. Raw metagenome read datasets for SRR7521238 and SRR8570466 were available in-house due to our affiliations with the respective data producers [15, 16, 34]. The library preparation protocols varied between these

datasets (Table 1). Adapter contaminants and low quality 3' ends were trimmed from the reads with Cutadapt v1.8.3 [24] using TrimGalore as a wrapper script [35]. The datasets of ERR526087, SRR5035895 and SRR7521238 were assembled using IDBA-UD [36]. The dataset of SRR8570466 was assembled with MegaHit [37] as described previously [15]. The assembly accompanying dataset SRS049959 in the abovementioned ftp site of the human metagenome project was used.

Quality-filtered sequencing reads were mapped to metagenome assemblies using bwa-mem v0.7.15-r1140 [30]. Following this, contigs shorter than 10 kb were discarded for reasons described above. Read depths in 500 nt sliding windows in each contig were calculated as described above. However, metagenome contigs larger than 10 kb were not subject to coverage-based filtering as each contig is treated as coming from an independent genetic element, and normalisation is performed within each contig (see below). This contrasts with the approach taken for the whole genome sequencing experiments where each contig passing all filtering steps is considered equally abundant. The difference in approach stems from the fact that too many contigs in metagenome assemblies will not have a chosen common GC-bin (e.g. 49%) and this would lead to severely reduced representation of contigs derived from genomes with high or low global GC contents. Within each metagenome contig, the 500 nt windows were binned by GC content into 1% wide bins and the average coverage of each 1% wide GC-bin was calculated within each contig. The coverage ratios of all pairwise combinations of GC-bins within each contig were then calculated (i.e. the coverage ratio is a ratio of the average coverage of a 1% wide numerator GC-bin to the average coverage of a 1% wide denominator GC-bin). Following this, the coverage ratio values

for each combination of two 1% wide GC-bins were averaged across all contigs that contain the relevant two GC-bins. These ratios were then log-transformed (base 10), such that values greater than zero indicated that metagenomic windows of the numerator's GC content are more covered than windows of the denominator's GC content and vice versa for values less than zero. These three dimensional data were plotted and rendered from a series of azimuth angles and elevations using the matplotlib and mpl_toolkits libraries of python. The images were saved in bitmap format, and the series of images were assembled, using ffmpeg V.3.4.2-2 (https://www.ffmpeg.org), into a video file to facilitate viewing of the plots in three dimensions. The pipelines to calculate coverage ratios between different metagenomics windows with different GC contents, along with source code for in-house scripts, is detailed in https://github.com/padbr/gcbias.

Quality of Illumina reads with respect to GC content

Raw Illumina reads were adapter trimmed with cutadapt (i) with quality filtering disabled, and (ii) with default quality filtering settings. Custom biopython scripts were used to evaluate the effects of quality filtering on the GC content of reads. The scripts calculated the GC content of each read and the median quality (Phred score) of each read within a dataset. The median quality values of reads of each GC content percentile were plotted using the boxplot function of matplotlib in python (Additional file 6). Furthermore, frequency distributions of the GC contents of reads with and without quality filtering were plotted using the hist function of matplotlib in python. Following this, relative proportions of reads for each GC content bin in the histogram were calculated by

dividing the proportions of the quality filtered reads by the corresponding proportions from the non-quality filtered reads (Additional file 7).

ddPCR

A pangenome analysis was performed, following the methods described in [38], on *Fusobacterium sp.* C1 and 18 other draft and complete *Fusobacterium* genomes (**Additional file 15**). From this, two single copy core genes were selected and primers targeting these and SSU rRNA were designed (Table 2). *Fusobacterium sp.* C1 genomic DNA was double digested with HindIII and DraI (NEB). ddPCR was performed to assess the ratio of SSU rRNA genes to two different single copy genes. ddPCR was performed using the QX-200 ddPCR system (Bio-Rad), using EvaGreen ddPCR Supermix. Data analyses were performed using QuantaSoft™ Analysis Pro software (Bio-Rad). Further details are available in **Additional file 2.**

Long range PCR product sequencing

Primers were designed to uniquely amplify two different 5.3 kb regions of the *Fusobacterium sp.* C1 genome with different GC contents: 30.2% (**Fig. 1, circle 3, green bar**) and 45.5% (**Fig. 1, circle 3, red bar**) (Table 3). Post amplification, the PCR products were quantified based on Qubit measurements and pooled into an equimolar mixture. Three independent paired PCR product mixtures were prepared in this manner (further details available in **Additional file 2**). Indexed libraries were prepared from

these pools using the Nextera XT kit and sequencing was performed on a MiSeq, as described for genome sequencing.

Availability of source code and requirements

Project name: gcbias

Project home page: https://github.com/padbr/gcbias

Operating system: Linux - probably Linux in general, but only tested with Ubuntu and CentOS

Programming language: python2.7, bash

Other requirements: bwa, samtools (>=1.0), ffmpeg, minimap2

License: MIT license

Any restrictions to use by non-academics: No restrictions

Availability of supporting data and materials

All sequencing reads associated with this project were deposited to SRA under BioProject accession number PRJNA503577. Data supporting this research are available in the *GigaScience* repository, GigaDB [39].

Declarations

List of abbreviations

HTS: high-throughput sequencing

SSU: small subunit

ddPCR: digital droplet PCR

Consent for publication

Not applicable

Competing interests

The authors declare that they have no competing interests

Funding

PDB was supported by a Villum Foundation Block Stipend. PDB and LHH were supported by a grant from the Danish Innovation Foundation (7076-00129B). TKN and LHH were supported by a grant (ORIGENE) from Aarhus University research fund (AUFF NOVA). MTPG was supported by a grant from the Danish National Advanced Technology Foundation (Højteknologifonden) (080-2012-3-Food genomics). None of the funding foundations played no role in the design of the study, the production, analysis and interpretation of the data nor in the writing of the manuscript.

Authors' contributions

The study was designed by LHH, TKN, WK and PDB. Lab work was performed by TKN, WK, MTPG, LP, MR, AA, and AZ. PDB, TKN, WK and LHH analysed the data. PDB wrote the paper. All authors revised the paper. All authors read and approved the final manuscript.

Acknowledgements

The authors thank Tina Thane and Tanja Begovic for technical assistance with DNA extractions and sequencing library preparations.

Additional files

Additional file 1

File name: Additional file 1.docx

Format: Microsoft Word; Extension: '.docx'

Title of data: Supplementary table 1: Genome sequencing data sets

A table describing which workflows were used to sequence which bacteria, and the accession numbers of each data set in the NCBI's sequence read archive.

Additional file 2

File name: Additional file 2.docx

Format: Microsoft Word; Extension: '.docx'

Title: Supplementary text: Supplementary methods and results

Description: Extra detail about the methods and results for the ddPCR analysis and extra information about the methods for filtering aberrantly covered contigs from analyses are included herein.

Additional file 3

File name: Additional file 3.docx

Format: Microsoft Word; Extension: '.docx'

Title: Supplementary figure 1

Description: Plots showing per-nucleotide coverage and GC content in 49 nt sliding windows and the positions of rRNA genes and protein coding genes from two 5.3 kb PCR products sequenced using the MiSeq workflow.

Additional file 4

File name: Additional file 4.docx

Format: Microsoft word; Extension: ‘.docx’

Title: Supplementary table 2: Numbers of reads mapped to two 5.3 kb equimolar PCR products from *Fusobacterium*

Description: The numbers of reads mapping to each of two 5.3 kb PCR products in each of three replicates are shown, along with a ratio indicating the relative coverage of each PCR product.

Additional file 5

File name: Additional file 5.docx

Format: Microsoft Word; Extension: '.docx'

Title: Supplementary figure 2

Description: Plots showing GC-biases in MiSeq and NextSeq workflows from several experiments along with quadratic lines of best fit.

Additional file 6

File name: Additional file 6.png

Format: png image; Extension: '.png'

Title: Supplementary figure 3

Description: For each dataset shown, the adapters were trimmed from the reads with quality filtering disabled. The read quality reads are represented in 1% wide GC-bins.

The orange dashes indicates the medians, the interquartile ranges are represented by boxes (rectangles) and the whiskers span the 10th to the 90th percentiles.

Additional file 7

File name: Additional file 7.png

Format: png image; Extension: '.png'

Title: Supplementary figure 4

Description: For each dataset shown, the adapters were trimmed from the reads both with and without quality filtering enabled. Histograms of the proportions of reads at various GC contents in each dataset were created, with identical bins of GC content for both datasets. These proportions for the quality filtered data were then divided by the proportions of the non-quality filtered data. In this way, it can be seen if quality filtering disproportionately affects the abundance of reads passing quality filtering if the ratio is significantly different to 1.0. Dark blue bars indicate that the GC-bin had at least 0.1% of the total abundance of reads in the dataset with quality filtering disabled, and below this value, the intensity of blue was scaled linearly down to no colour. This colour scaling focuses attention on the GC contents that are reasonably abundant in the 500 nt windows in the genomic GC-bias analyses.

Additional file 8

File name: Additional file 8.mp4

Format: VLC media player; Extension: '.mp4'

Title: Supplementary video 1

Description: GC-bias in female human faecal metagenome (SRA acc. no. ERR526087). Movie file showing log-transformed (base 10) average coverage of 500 nt-windows of a foreground GC content divided by the average coverage of 500 nt-windows of a background GC content.

Additional file 9

File name: Additional file 9.mp4

Format: VLC media player; Extension: '.mp4'

Title: Supplementary video 2

Description: GC-bias in kelp associated biofilm metagenome (SRA acc. no. SRR5035895). Movie file showing log-transformed (base 10) average coverage of 500 nt-windows of a foreground GC content divided by the average coverage of 500 nt-windows of a background GC content.

Additional file 10

File name: Additional file 10.mp4

Format: VLC media player; Extension: '.mp4'

Title: Supplementary video 3

Description: GC-bias in human male faecal metagenome (SRA acc. no. SRS049959). Movie file showing log-transformed (base 10) average coverage of 500 nt-windows of a foreground GC content divided by the average coverage of 500 nt-windows of a background GC content.

Additional file 11

File name: Additional file 11.mp4

Format: VLC media player; Extension: '.mp4'

Title: Supplementary video 4

Description: GC-bias in moving bed biofilm reactors with effluent wastewater metagenome (SRA acc. no. SRR8570466). Movie file showing log-transformed (base 10) average coverage of 500 nt-windows of a foreground GC content divided by the average coverage of 500 nt-windows of a background GC content.

Additional file 12

File name: Additional file 12

Format: VLC media player; Extension: '.mp4'

Title: Supplementary video 5

Description: GC-bias in turkey vulture intestinal contents metagenome (SRA acc. no. SRR7521238). Movie file showing log-transformed (base 10) average coverage of 500

nt-windows of a foreground GC content divided by the average coverage of 500 nt-windows of a background GC content.

Additional file 13

File name: Additional file 13.docx

Format: Microsoft Word; Extension: '.docx'

Title: Supplementary figure 5

Description: Histogram showing GC content of SSU rRNA genes in the greengenes database

Additional file 14

File name: Additional file 14.png

Format: Bitmap image, '.png'

Title: Supplementary figure 6

Description: All results presented in figures 2-3 were repeated for a range of different genomic window sizes ranging from 50 nt to 5000 nt. The methodology was the same as presented in figures 2-3, except that the coverage values were not normalized to the coverage of windows with 49% GC, as this was not feasible. Instead, the coverage was normalized according to the average coverage in each dataset.

Additional file 15

File name: Additional file 15.docx

Format: Microsoft Excel; Extension: '.xlsx'

Title: Supplementary table 3: Genome sequences used to identify single copy genes in *Fusobacterium*

Description: Accession numbers used in a comparative genomics approach which identified genes as single-copy core genes in the *Fusobacterium* genus. Two of these single-copy core genes were selected as targets for the ddPCR experiment.

References

1. Reuter Jason A, Spacek DV and Snyder Michael P. High-throughput sequencing technologies. Molecular Cell. 2015;58 4:586-97. doi:10.1016/j.molcel.2015.05.004.
2. Schirmer M, Ijaz UZ, D'Amore R, Hall N, Sloan WT and Quince C. Insight into biases and sequencing errors for amplicon sequencing with the Illumina MiSeq platform. Nucleic Acids Res. 2015;43 6:e37. doi:10.1093/nar/gku1341.
3. Brooks JP, Edwards DJ, Harwich MD, Rivera MC, Fettweis JM, Serrano MG, et al. The truth about metagenomics: quantifying and counteracting bias in 16S rRNA studies. BMC Microbiol. 2015;15 1:66. doi:10.1186/s12866-015-0351-6.
4. Jakobsen TH, Hansen MA, Jensen PØ, Hansen L, Riber L, Cockburn A, et al. Complete genome sequence of the cystic fibrosis pathogen *Achromobacter xylosoxidans* NH44784-1996 complies with important pathogenic phenotypes. PLoS One. 2013;8 7:e68484. doi:10.1371/journal.pone.0068484.
5. Quail MA, Smith M, Coupland P, Otto TD, Harris SR, Connor TR, et al. A tale of three next generation sequencing platforms: comparison of Ion Torrent, Pacific Biosciences and Illumina MiSeq sequencers. BMC Genomics. 2012;13 1:341. doi:10.1186/1471-2164-13-341.
6. Oyola SO, Otto TD, Gu Y, Maslen G, Manske M, Campino S, et al. Optimizing illumina next-generation sequencing library preparation for extremely at-biased genomes. BMC Genomics. 2012;13 1:1. doi:10.1186/1471-2164-13-1.
7. van Dijk EL, Jaszczyszyn Y and Thermes C. Library preparation methods for next-generation sequencing: Tone down the bias. Experimental Cell Research. 2014;322 1:12-20. doi:http://dx.doi.org/10.1016/j.yexcr.2014.01.008.

8. Jones MB, Highlander SK, Anderson EL, Li W, Dayrit M, Klitgord N, et al. Library preparation methodology can influence genomic and functional predictions in human microbiome research. Proceedings of the National Academy of Sciences. 2015;112 45:14024-9. doi:10.1073/pnas.1519288112.
9. Ross MG, Russ C, Costello M, Hollinger A, Lennon NJ, Hegarty R, et al. Characterizing and measuring bias in sequence data. Genome Biol. 2013;14 5:R51. doi:10.1186/gb-2013-14-5-r51.
10. Chen Y-C, Liu T, Yu C-H, Chiang T-Y and Hwang C-C. Effects of GC bias in next-generation-sequencing data on de novo genome assembly. PLoS One. 2013;8 4:e62856. doi:10.1371/journal.pone.0062856.
11. Benjamini Y and Speed TP. Summarizing and correcting the GC content bias in high-throughput sequencing. Nucleic Acids Res. 2012;40 10:e72. doi:10.1093/nar/gks001.
12. Quail MA, Kozarewa I, Smith F, Scally A, Stephens PJ, Durbin R, et al. A large genome centre’s improvements to the Illumina sequencing system. Nat Methods. 2008;5 12:1005-10. doi:10.1038/nmeth.1270.
13. Aird D, Ross MG, Chen W-S, Danielsson M, Fennell T, Russ C, et al. Analyzing and minimizing PCR amplification bias in Illumina sequencing libraries. Genome Biol. 2011;12 2:R18-R. doi:10.1186/gb-2011-12-2-r18.
14. Chitsaz H, Yee-Greenbaum JL, Tesler G, Lombardo M-J, Dupont CL, Badger JH, et al. De novo assembly of bacterial genomes from single cells. Nat Biotechnol. 2011;29 10:915-21. doi:10.1038/nbt.1966.
15. Escolà Casas M, Nielsen TK, Kot W, Hansen LH, Johansen A and Bester K. Degradation of mecoprop in polluted landfill leachate and waste water in a moving bed biofilm reactor. Water Research. 2017;121:213-20. doi:https://doi.org/10.1016/j.watres.2017.05.031.
16. Roggenbuck M, Bærholm Schnell I, Blom N, Bælum J, Bertelsen MF, Sicheritz-Pontén T, et al. The microbiome of New World vultures. Nature Communications. 2014;5:5498. doi:10.1038/ncomms6498

http://www.nature.com/articles/ncomms6498#supplementary-information.
17. DeSantis TZ, Hugenholtz P, Larsen N, Rojas M, Brodie EL, Keller K, et al. Greengenes, a chimera-checked 16S rRNA gene database and workbench compatible with ARB. Appl Environ Microb. 2006;72 7:5069-72. doi:10.1128/aem.03006-05.
18. Edgar RC. UNBIAS: An attempt to correct abundance bias in 16S sequencing, with limited success. bioRxiv. 2017; doi:10.1101/124149.
19. Deamer D, Akeson M and Branton D. Three decades of nanopore sequencing. Nat Biotechnol. 2016;34:518. doi:10.1038/nbt.3423.
20. Head SR, Komori HK, LaMere SA, Whisenant T, Van Nieuwerburgh F, Salomon DR, et al. Library construction for next-generation sequencing: overviews and challenges. Biotechniques. 2014;56 2:61-passim. doi:10.2144/000114133.
21. Bowers RM, Clum A, Tice H, Lim J, Singh K, Ciobanu D, et al. Impact of library preparation protocols and template quantity on the metagenomic reconstruction of a mock microbial community. BMC Genomics. 2015;16 1:856. doi:10.1186/s12864-015-2063-6.

22. Meyer M and Kircher M. Illumina sequencing library preparation for highly multiplexed target capture and sequencing. Cold Spring Harbor Protocols. 2010;2010 6:pdb.prot5448. doi:10.1101/pdb.prot5448.
23. Nielsen TK, Rasmussen M, Demanèche S, Cecillon S, Vogel TM and Hansen LH. Evolution of sphingomonad gene clusters related to pesticide catabolism revealed by genome sequence and mobilomics of *Sphingobium herbicidovorans* MH. Genome Biol Evol. 2017;9 9:2477-90. doi:10.1093/gbe/evx185.
24. Martin M. Cutadapt removes adapter sequences from high-throughput sequencing reads. EMBnetjournal. 2011;17 1:10-2. doi:10.14806/ej.17.1.200.
25. Chaisson MJ and Tesler G. Mapping single molecule sequencing reads using basic local alignment with successive refinement (BLASR): application and theory. BMC Bioinformatics. 2012;13 1:238. doi:10.1186/1471-2105-13-238.
26. Lindgreen S. AdapterRemoval: easy cleaning of next-generation sequencing reads. BMC Research Notes. 2012;5 1:337. doi:10.1186/1756-0500-5-337.
27. Bankevich A, Nurk S, Antipov D, Gurevich AA, Dvorkin M, Kulikov AS, et al. SPAdes: A new genome assembly algorithm and its applications to single-cell sequencing. Journal of Computational Biology. 2012;19 5:455-77. doi:10.1089/cmb.2012.0021.
28. Aziz RK, Bartels D, Best AA, DeJongh M, Disz T, Edwards RA, et al. The RAST server: Rapid annotations using subsystems technology. BMC Genomics. 2008;9:75-. doi:10.1186/1471-2164-9-75.
29. Alikhan N-F, Petty NK, Ben Zakour NL and Beatson SA. BLAST Ring Image Generator (BRIG): simple prokaryote genome comparisons. BMC Genomics. 2011;12 1:1-10. doi:10.1186/1471-2164-12-402.
30. Li H. Aligning sequence reads, clone sequences and assembly contigs with BWA-MEM. 2013.
31. Li H. Minimap2: pairwise alignment for nucleotide sequences. ArXiv e-prints. 2017.
32. Li H, Handsaker B, Wysoker A, Fennell T, Ruan J and Homer N. The sequence alignment/map format and SAMtools. Bioinformatics. 2009;25 doi:10.1093/bioinformatics/btp352.
33. Iglewicz B and Hoaglin DC. How to detect and handle outliers. ASQC Quality Press; 1993.
34. Zepeda Mendoza ML, Roggenbuck M, Manzano Vargas K, Hansen LH, Brunak S, Gilbert MTP, et al. Protective role of the vulture facial skin and gut microbiomes aid adaptation to scavenging. Acta Veterinaria Scandinavica. 2018;60 1:61. doi:10.1186/s13028-018-0415-3.
35. Krueger F: Trim Galore! http://www.bioinformatics.babraham.ac.uk/projects/trim_galore/.
36. Peng Y, Leung HCM, Yiu SM and Chin FYL. IDBA-UD: a de novo assembler for single-cell and metagenomic sequencing data with highly uneven depth. Bioinformatics. 2012;28 11:1420-8. doi:10.1093/bioinformatics/bts174.
37. Li D, Luo R, Liu C-M, Leung C-M, Ting H-F, Sadakane K, et al. MEGAHIT v1.0: A fast and scalable metagenome assembler driven by advanced methodologies and community practices. Methods. 2016;102:3-11. doi:https://doi.org/10.1016/j.ymeth.2016.02.020.

38. Browne P, Tamaki H, Kyrpides N, Woyke T, Goodwin L, Imachi H, et al. Genomic composition and dynamics among *Methanomicrobiales* predict adaptation to contrasting environments. ISME J. 2017;11 1:87-99. doi:10.1038/ismej.2016.104.
39. Browne PD, Nielsen TK, Kot W, Aggerholm A, Gilbert MTP, Puetz L, et al. Supporting data for "Substantial GC-bias impacts genomic and metagenomic reconstructions, significantly underrepresenting GC-poor organisms". GigaScience Database. 2020; doi:http://dx.doi.org/10.5524/100696.
40. Bäckhed F, Roswall J, Peng Y, Feng Q, Jia H, Kovatcheva-Datchary P, et al. Dynamics and stabilization of the human gut microbiome during the first year of life. Cell Host & Microbe. 2015;17 5:690-703. doi:https://doi.org/10.1016/j.chom.2015.04.004.
41. Vollmers J, Frentrup M, Rast P, Jogler C and Kaster A-K. Untangling genomes of novel planctomycetal and verrucomicrobial species from Monterey Bay kelp forest metagenomes by refined binning. Front Microbiol. 2017;8:472. doi:10.3389/fmicb.2017.00472.

Figures and tables

Tables

Table 1: Sources of datasets for GC-bias analysis in metagenome sequencing

Accession no. / Name (Relevant supplementary data)	Sequencing technology	Library preparation kit	Environment	Reference	Total Contigs > 10 kb	Assembly length > 10 kb	N_{50} > 10 kb	Num. PCR cycles
ERR526087 (Additional file 8)	HiSeq 2000	Paired-End Genomic DNA Sample Prep Kit (Illumina)	Human faeces (female)	[40]	2880	71.9 Mb	29679	10 – 12

SRR5035895 (Additional file 9)	MiSeq	NEBnext Ultra	Kelp associated biofilm	[41]	217	3.77 Mb	18496	4 – 12
SRS049959 (Additional file 10)	GA II	Paired-End Genomic DNA Sample Prep Kit (Illumina)	Human faeces (male)	NIH Human Microbiome Project	1409	21.6 Mb	14775	10 – 12
SRR8570466 (Additional file 11)	NextSeq	Nextera	Moving bed biofilm reactors with effluent wastewater	[15]	5496	109 Mb	20186	8
SRR7521238 (Additional file 12)	HiSeq 2500	NEBNext	Intestinal contents of a turkey vulture	[34]	1256	26.9 Mb	22974	14

Assembly statistics are presented for contigs larger than 10 kb only. The number of PCR cycles used during library preparation was inferred from the library preparation kit's instructions when it couldn't be found in the referenced publications.

Table 2: Primer pairs used for ddPCR

Product	Forward primer	Reverse primer	Product size
ATP synthase β-subunit	TGCTAAGGGACATGGAGGAC	AAGTCATCGGCTGGTACGTA	414 bp

SSU ribosomal protein S3	CGGAAGAAAAGGTGCTGAAAT	CTACGCTTCTCCTCCTTCCC	424 bp
SSU ribosomal RNA	GCAGCAGTGGGGAATATTGG	CTGTTTGCTACCCACGCTTT	413 bp

Table 3: Primers used to amplify 5.3 kb regions with different GC contents from *Fusobacterium* C1's genome

Primer name	Primer Sequence	Orientation	Region
NormA_F	TACTAGCTCCACTTTTAATACCTG	fwd	1350019..1350042
NormA_R	GCTCTTCTTATTTCACCTTCATCT	rev	complement(1355348..1355371)
RNA_F	CTGTCTTTGCAAACCTTTCTATT	fwd	1317778..1317800
RNA_R	ATTTGGCTTCTTGTGTTTTAGTT	rev	complement(1323108..1323130)

Figures

Figure 1: Coverage biases in the sequencing of *Fusobacterium sp*. C1. The circle plot shows from the inside: GC content (Ring 1), positions of CDSs, rRNAs, and tRNAs (Ring 2), positions of the PCR targets for ddPCR and the 5.3 kb PCR products (Ring 3), and coverages of Nanopore reads, MiSeq reads, NextSeq reads, HiSeq reads and PacBio reads (Rings 4 – 8 respectively). The circles are numbered from the inside. The

GC content plot is centred on the median GC content, with GC contents greater than the median extending outwards. The coverage data is plotted in 50 nt windows, with separate linear scales for each dataset.

Figure 2: Coverage biases in MiSeq datasets from many bacteria with different GC contents. Dot plots show local GC content and normalised relative coverages in 500 nt windows (see methods for explanation) of MiSeq data from a variety of bacteria with different average GC contents. Error bars indicate ± one standard deviation of normalised coverage. The intensity of the blue in the dots is a log-transformed heatmap of the number of 500 nt windows averaged into that datapoint. The datapoint with the most windows in each plot has maximum blue. The vertical green line marks the average GC content of each assembly. The average normalised coverage value is indicated with a horizontal dashed red line.

Figure 3: GC-biases in NextSeq, PacBio, Nanopore and HiSeq data. The dot plots are as described in Figure 2.

Figure 1

Click here to access/download;Figure;Fig1.tif

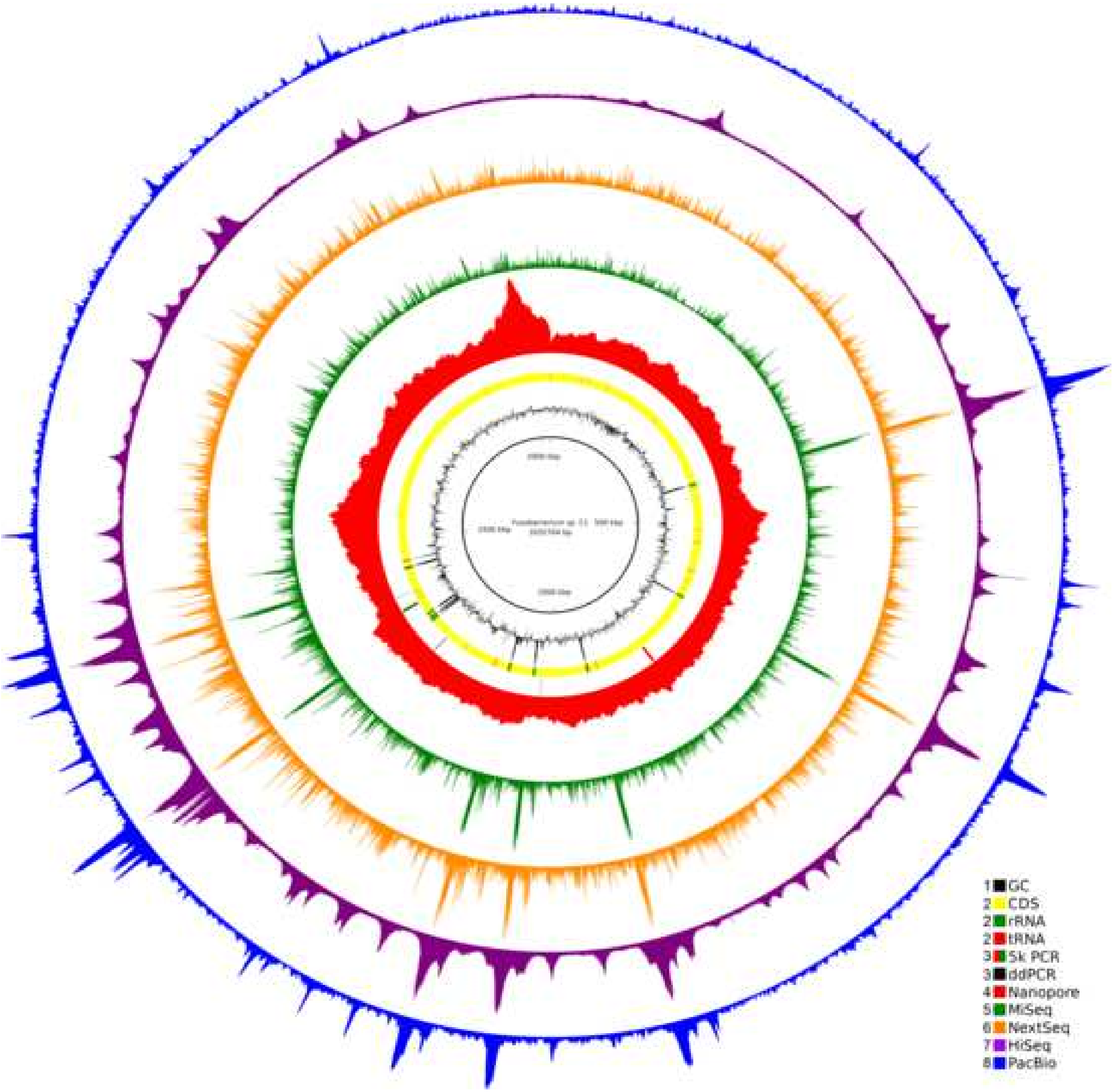

Figure 2

Click here to access/download;Figure;Fig2_MiSeq.tif

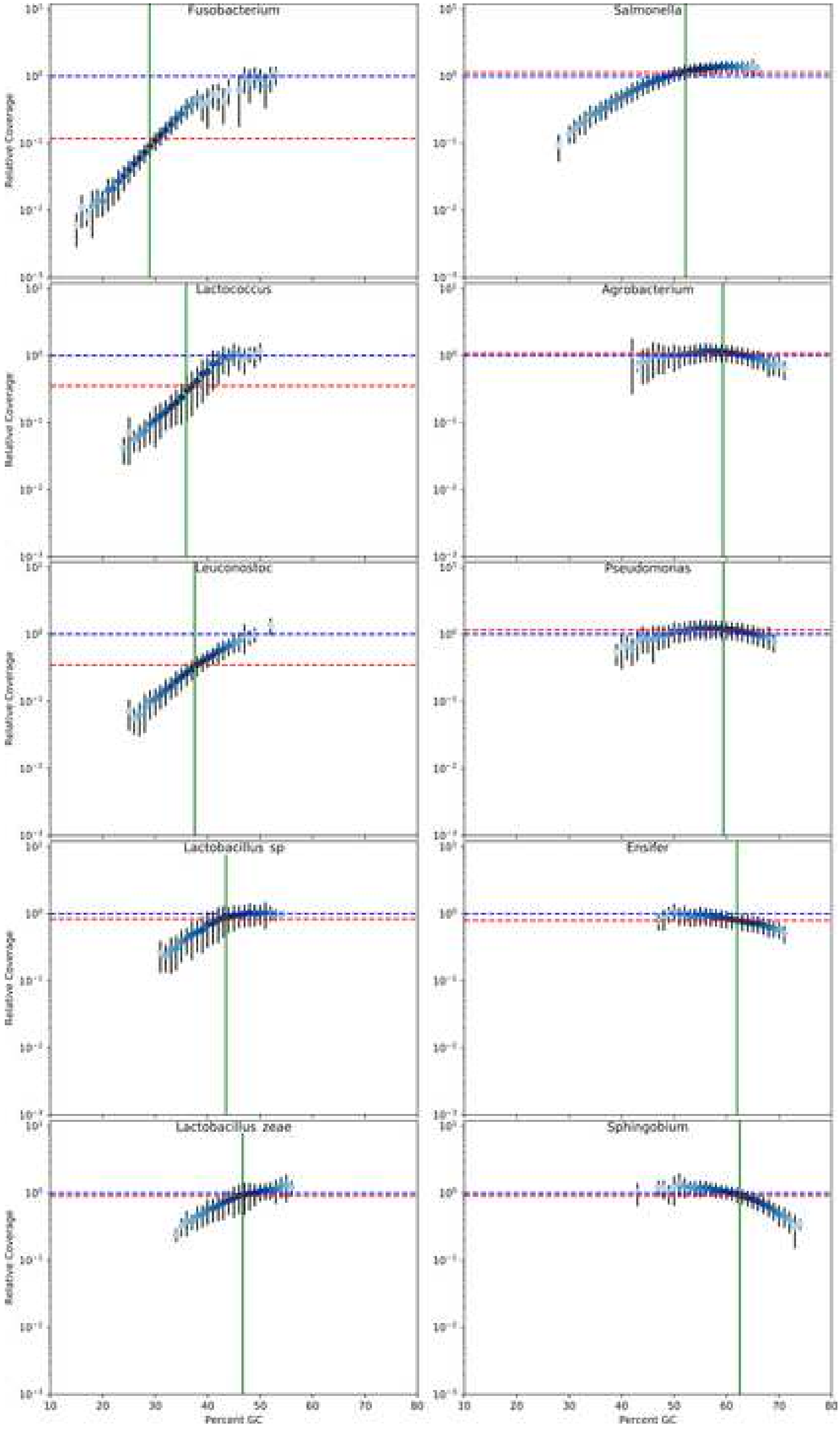

Figure 3

Click here to access/download;Figure;Fig3_Others.tif

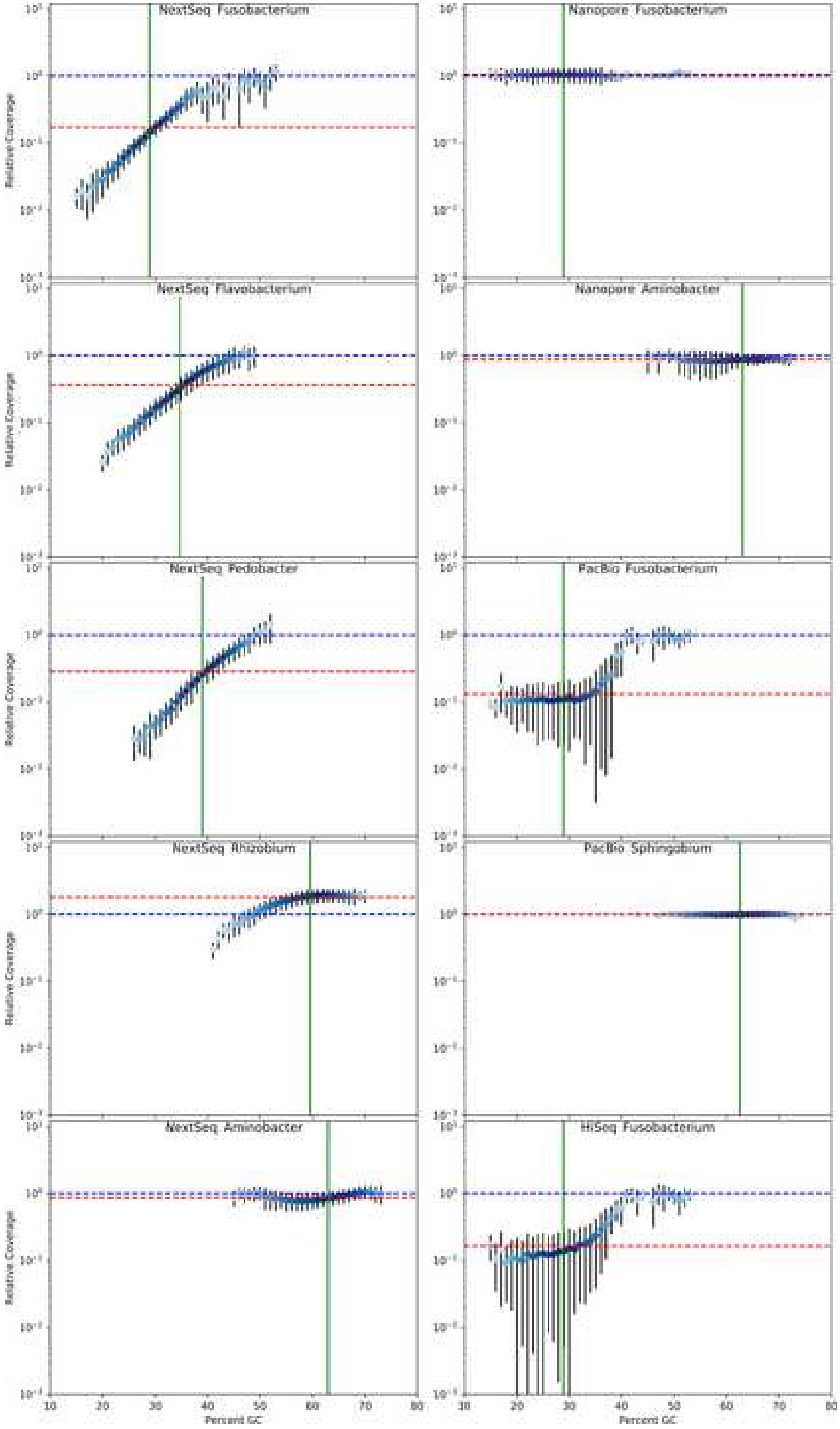

Additional file 1

Click here to access/download
Supplementary Material
Additional file 1.docx

Additional file 2

Click here to access/download
Supplementary Material
Additional file 2.docx

Additional file 3

Click here to access/download
Supplementary Material
Additional file 3.docx

Click here to access/download
Supplementary Material
Additional file 4.docx

Click here to access/download
Supplementary Material
Additional file 5.docx

Additional file 6

Click here to access/download
Supplementary Material
Additional file 6.png

Additional file 7

Click here to access/download
Supplementary Material
Additional file 7.png

Additional file 8

Click here to access/download
Supplementary Material
Additional file 8.mp4

Additional file 9

Click here to access/download
Supplementary Material
Additional file 9.mp4

Click here to access/download
Supplementary Material
Additional file 10.mp4

Additional file 11

Click here to access/download
Supplementary Material
Additional file 11.mp4

Click here to access/download
Supplementary Material
Additional file 12.mp4

Additional file 13

Click here to access/download
Supplementary Material
Additional file 13.docx

Additional file 14

Click here to access/download

Supplementary Material

Additional file 14.png

Additional file 15

Click here to access/download
Supplementary Material
Additional file 15.docx
