# Supplementary material for: GC bias affects genomic and metagenomic reconstructions, underrepresenting GC-poor organisms
Source: Gigascience. 2020 Feb 13;9(2):giaa008. doi: 10.1093/gigascience/giaa008 (PMC7016772; doi:10.1093/gigascience/giaa008)
Supplement: giaa008_Supplemental_Files [file giaa008_supplemental_files.zip › Additional file 1.docx]

**Supplementary Table 1** Genome sequencing data sets.

| Workflow | Bacterium | GC content | No. Reads | Read length | BioProject | BioSample | Accession |
| --- | --- | --- | --- | --- | --- | --- | --- |
| MiSeq | *Fusobacterium sp* C1 | 28.9 | 713019 | 2x251 | PRJNA503577 | SAMN10485398 | SRR8257184 |
| MiSeq | *Lactotoccus lactis* | 35.9 | 852988 | 2x301 | PRJNA503577 | SAMN10762653 | SRR8450504 |
| MiSeq | *Leuconostoc mesenteroides* | 37.5 | 1703156 | 2x251 | PRJNA503577 | SAMN05981503 | SRR8450599 |
| MiSeq | *Lactobacillus sp* | 43.5 | 837911 | 2x251 | PRJNA503577 | SAMN10762815 | SRR8474138 |
| MiSeq | *Lactobacillus zeae* | 46.7 | 602435 | 2x251 | PRJNA503577 | SAMN10762816 | SRR8450684 |
| MiSeq | *Salmonella enterica* | 52.2 | 1489561 | 2x251 | PRJNA503577 | SAMN10764971 | SRR8451733 |
| MiSeq | *Agrobacterium sp* AS1YR2 | 59.3 | 252359 | 2x251 | PRJNA503577 | SAMN10764955 | SRR8474136 |
| MiSeq | *Pseudomonas sp* | 59.4 | 215622 | 2x251 | PRJNA503577 | SAMN10764959 | SRR8472151 |
| MiSeq | *Ensifer adhaerens* AS2PG1 | 62.0 | 175193 | 2x251 | PRJNA503577 | SAMN10364938 | SRR8156493 |
| MiSeq | *Sphingobium herbicidovorans* MH | 62.5 | 352654 | 2x251 | PRJNA503577 | SAMN06647797 | SRR8570467 |
| NextSeq | *Fusobacterium sp* C1 | 28.9 | 4075337 | 2x151 | PRJNA503577 | SAMN10485398 | SRR8257183 |
| NextSeq | *Flavobacterium succinicans* GA1LYS1 | 34.7 | 16588561 | 2x151 | PRJNA503577 | SAMN10364939 | SRR8156492 |
| NextSeq | *Pedobacter cryoconitis* GB1LGT1 | 39.0 | 19972340 | 2x151 | PRJNA503577 | SAMN10364940 | SRR8156494 |
| NextSeq | *Rhizobium sp* GC2LW1 | 59.5 | 11631123 | 2x151 | PRJNA503577 | SAMN10765043 | SRR8474137 |
| NextSeq | *Aminobacter sp* MSH1 | 63.0 | 8467701 | 2x151 | PRJNA503577 | SAMN10765146 | SRR8472147 |
| HiSeq | *Fusobacterium sp* C1 | 28.9 | 14717431 | 2x101 | PRJNA503577 | SAMN10485398 | SRR8257185 |
| Nanopore | *Fusobacterium sp* C1 | 28.9 | 10347 | 173,  9199, 30731,  161230 | PRJNA503577 | SAMN10485398 | SRR8451906 |
| Nanopore | *Aminobacter sp* MSH1 | 63.0 | 6900 | 192,  7566, 33472,  199385 | PRJNA503577 | SAMN10765146 | SRR8472146 |
| PacBio | *Fusobacterium sp* C1 | 28.9 | 17333 | 501,  3224,  2147,  20128 | PRJNA503577 | SAMN10485398 | SRR8570474 |
| PacBio | *Sphingobium herbicidovorans* MH | 62.5 | 10894 | 1121,  8799.5,  10784,  35708 | PRJNA503577 | SAMN06647797 | SRR8570539 |

For each single genome sequencing dataset used in this work, the workflow used, the bacterium sequenced, its average GC content, the number of reads and the read lengths are shown. Also shown are the accession numbers for the datasets uploaded to the SRA. The read lengths for the Nanopore and PacBio workflows indicate the minimum, median, N50 and maximum read lengths.
