# Supplementary material for: GC bias affects genomic and metagenomic reconstructions, underrepresenting GC-poor organisms
Source: Gigascience. 2020 Feb 13;9(2):giaa008. doi: 10.1093/gigascience/giaa008 (PMC7016772; doi:10.1093/gigascience/giaa008)
Supplement: giaa008_Supplemental_Files [file giaa008_supplemental_files.zip › Additional file 13.docx]

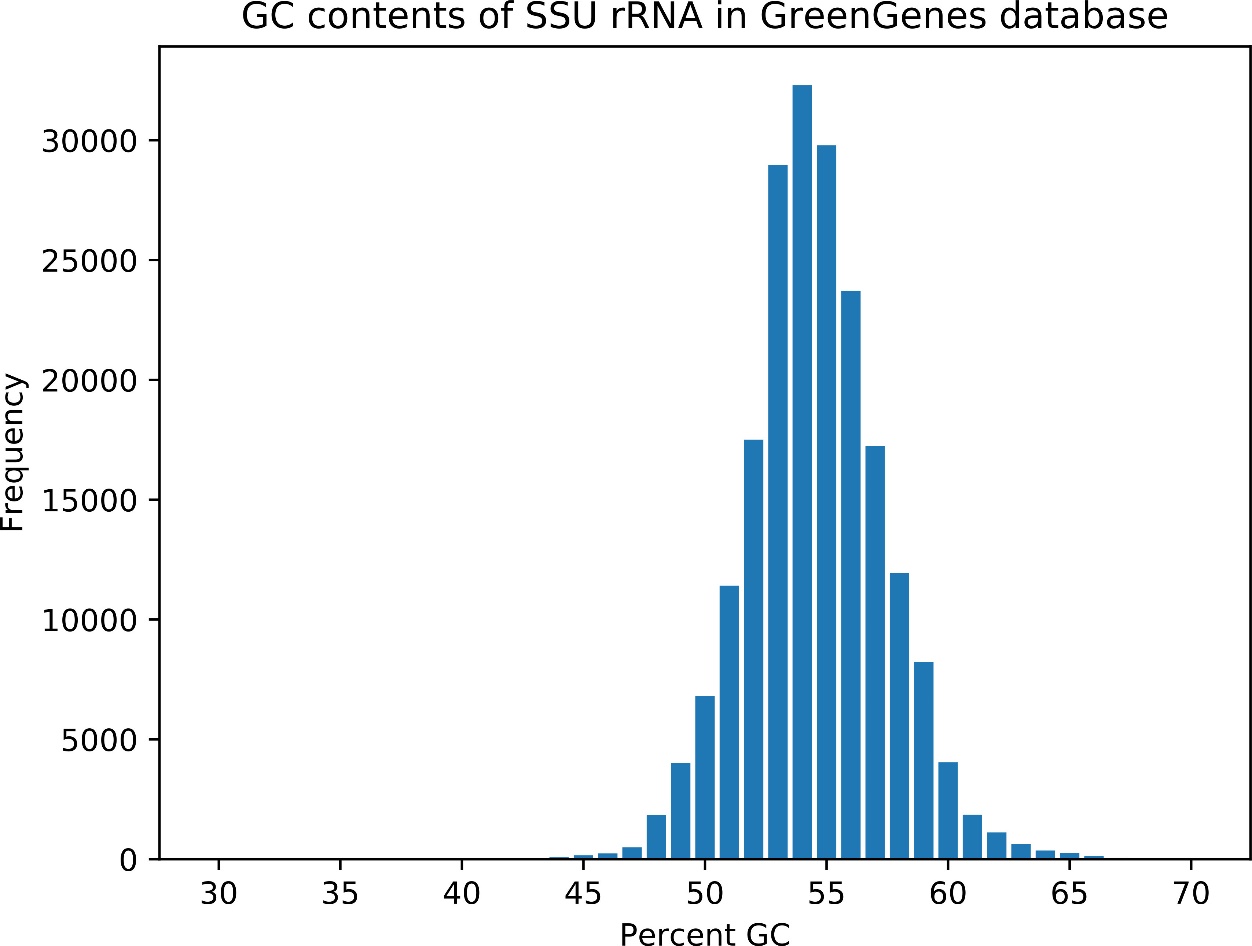


**Supplementary Figure 5** Histogram showing GC content of SSU rRNA genes in the greengenes database
