# Supplementary material for: GC bias affects genomic and metagenomic reconstructions, underrepresenting GC-poor organisms
Source: Gigascience. 2020 Feb 13;9(2):giaa008. doi: 10.1093/gigascience/giaa008 (PMC7016772; doi:10.1093/gigascience/giaa008)
Supplement: giaa008_Supplemental_Files [file giaa008_supplemental_files.zip › Additional file 15.docx]

**Supplementary Table 3** Genome sequences used to identify single copy genes in *Fusobacterium*

| Name | Number of contigs | Accession number(s) |
| --- | --- | --- |
| Fusobacterium ulcerans ATCC 49185 | 8 | ACDH02000000 |
| Fusobacterium nucleatum subsp. nucleatum | 3 | LMVH01000000 |
| Fusobacterium necrophorum subsp. funduliforme B35 | 40 | AOJP01000000 |
| Fusobacterium mortiferum ATCC 9817 | 8 | ACDB02000000 |
| Fusobacterium ulcerans 12-1B | 8 | AGWJ02000000 |
| Fusobacterium nucleatum subsp. animalis | 1 | CP012715 |
| Fusobacterium nucleatum subsp. polymorphum | 3 | CP013121, CP013122, CP013123 |
| Fusobacterium equinum | 77 | LRPX01000000 |
| Fusobacterium varium ATCC 27725 | 11 | ACIE02000000 |
| Fusobacterium periodonticum D10 | 150 | ACIF01000000 |
| Fusobacterium necrophorum DJ-2 | 226 | JAAH01000000 |
| Fusobacterium nucleatum subsp. animalis ATCC 51191 | 465 | AFQD01000000 |
| Fusobacterium hwasookii ChDC F300 | 2 | CP013334, CP013335 |
