# Supplementary material for: GC bias affects genomic and metagenomic reconstructions, underrepresenting GC-poor organisms
Source: Gigascience. 2020 Feb 13;9(2):giaa008. doi: 10.1093/gigascience/giaa008 (PMC7016772; doi:10.1093/gigascience/giaa008)
Supplement: giaa008_Supplemental_Files [file giaa008_supplemental_files.zip › Additional file 2.docx]

# Supplementary text: Supplementary methods and results

## Supplementary methods

### Identification of contigs with abnormal coverage

Following assembly (detailed in the main text’s methods section), contigs less than 10 kb in length were removed, since they will have a low number of sliding windows, and may be associated with complications in assembly (e.g. unresolved repeats) and other non-chromosomal entities (including mobile genetic elements). Because the elimination of small contigs does not fully remove the chances of plasmids, phages, repeats etc. being in the assembly, contigs greater than 10 kb with abnormal coverage were identified and removed from the coverage evenness assessments presented in the main article. The identification of contigs with abnormal coverage was done as follows:

After eliminating contigs smaller than 10 kbp, read depths (calculated by using samtools (‘depth –a’ option)) at each position of a contig’s sequence were summed and divided by the number of nucleotides in the contig to get an average coverage value for each contig. The average coverages of contigs were handled as a list of floating point numbers in python (assigned to a variable named points) as detailed in the following python code, taken almost verbatim from <https://github.com/joferkington/oost_paper_code/blob/master/utilities.py>, which is itself based on a method described elsewhere ^[1](#_ENREF_1)^:

import numpy as np

def is_outlier(points, thresh=10):

points = np.array(points)

points = points[:,None]

median = np.median(points, axis=0)

diff = np.sum((points – median)**2, axis = -1)

diff = np.sqrt(diff)

med_abs_deviation = np.median(diff)

modified_z_score = 0.6745 * diff / med_abs_deviation

return modified_z_score > thresh

In algebraic terms, a modified z-score was calculated as follows:

n = number of contigs longer than 10 kb

C_i_ = average coverage of the i^th^ contig longer than 10 kb

M­_C_ = median {C_1_, C_2_, …, C_n-1_, C_n_}

D_i_ = √((C_i_ – M_C_)^2^)

M_D_ = median {D_1_, D_2_, …, D_n-1_, D_n_}

Z_i_ = 0.6745*D_i_/M_D_

Contigs with a modified z-score greater than a critical threshold (10 in this case) were treated as abnormally covered and not included in assessments of GC-bias.

The method was supervised by examining a 3D plot of contig length versus contig coverage versus contig GC content, with normal coverage and abnormal coverage discriminated by colour. This supervision was to intuitively assess whether or not contigs were being called as abnormally covered due to extremes in GC content. In no case was there any indication that long contigs (> 10 kb) were called as abnormally covered due to extremes in GC content. This is fully detailed in a github repository associated with this article (https://github.com/padbr/gcbias).

### ddPCR

*Fusobacterium sp.* C1 was grown for 24 hours at 37 ^O^C on LB agar plates, reduced with 0.05% w/v cysteine under a nitrogen atmosphere. It was scraped into reduced LB broth with 15% w/v glycerol and stored at -80 ^O^C for 2 months. Genomic DNA (gDNA) was isolated using the Genomic Mini AX Bacteria kit (A&A Biotechnology). 0.5 μg of gDNA was diluted to 300 μl in 1X Cutsmart buffer (NEB) and digested simultaneously with HindIII and DraI (NEB) at 37 ^O^C for 18 hours. The digested DNA was purified and recovered in 10 μl EB (Qiagen) after an ethanol precipitation^[2](#_ENREF_2)^. The digestion efficacy was confirmed with agarose gel electrophoresis using an undigested aliquot of gDNA as a reference. DNA concentrations were quantified using a Qubit HS dsDNA kit (Thermo Fisher). Digital droplet PCR (ddPCR) was used to assess the ratio of small subunit rRNA (SSU rRNA) genes to two single copy genes, an ATP synthase beta subunit and an SSU ribosomal protein S3 with the primers listed in table 2 (main text). Orthologs of these two single copy genes were also present in single copy in 18 other publicly available (draft) *Fusobacterium* genome sequences. In each 20 µl PCR reaction mixture 10 µl 2x EvaGreen ddPCR Supermix (Bio-Rad), primers at a final concentration of 0.1 µM and template at either 6,250 or 12,500 genome copies were included. A no template control was performed for each assay. Each reaction mixture was partitioned into approximately 20,000 droplets with a QX-200 droplet generator (Bio-Rad). PCR amplification was performed with the following conditions: 95 °C for 5 min (1 cycle); 95 °C for 30 s, 58 °C for 30 s and 72 °C for 1 min (40 cycles); 4 °C for 5 min (1 cycle); 90 °C for 5 min (1 cycle); 4 °C hold. Droplets were read individually with the QX200 droplet reader (Bio-Rad). Data analysis was performed using QuantaSoft™ Analysis Pro software (Bio-Rad).

## Supplementary Results

### Coverage biases investigated in normalised long range PCR products

Two 5.4 kbp regions of the *Fusobacterium* *sp.* C1 genome, with different (30.2% vs 45.5%) GC contents were amplified by PCR. The products were quantified using a Qubit 2.0 Fluorometer (Thermo Fisher) and mixed in equimolar ratios. Nextera XT libraries were prepared and the PCR products were sequenced on the MiSeq platform. This was done in triplicate. After sequencing, the reads were mapped to the sequences of the PCR products, and numbers of mapped reads were counted.

The two regions, inclusive of primer sequences, are:

CDS locus – 30.2% GC fragment, 5353 bp, chr1:1350019-1355371

RNA locus – 45.5% GC fragment, 5353 bp, dhr1:1317778-1323130

The RNA locus (45.5% GC) to the CDS locus (30.2% GC) coverage ratios varied considerably between the replicates. A two-tailed t-test (libreoffice calc, paired type) gave a P-value of ca. 0.015, indicating that the CDS locus and RNA locus had significantly different coverage levels. However, the variation in the ratios (Additional file 4: right column) is high.

Coverage plots were made to assess if any artefacts could be causing the discrepancy in coverage ratios. The RNA locus (optimal GC) evidenced high coverage, with dips in coverage at the beginning, end and between 16S and 23S (Additional file 3). The coverage profiles of the CDS locus (suboptimal GC) also had similar “shapes”, and replicate 2 (blue trace) had lower coverage overall (Additional file 3). The regions around 16S and 23S have lower GC contents than the 16S and 23S genes themselves. The low coverage in these areas could also be due to proximity to the ends of the DNA molecules. However, the low coverage evident between SSU rRNA and LSU rRNA corresponding to a dip in GC content is a striking demonstration of local GC content being correlated with coverage bias in the Nextera XT/MiSeq workflow. Overall, the coverage depth profiles seem to correspond to the GC content profiles.

It is also noteworthy that one of the replicates (no. 2) gave a much higher ratio (10.63) of the RNA locus to the CDS locus (4.14 and 5.39). It is the case the replicate no. 2 had less coverage than the other two replicates. However, the similar trends between replicate 2 and replicates 1 and 3 in Additional file 4 indicate that there was simply a different ratio in replicate 2 than in replicates 1 and 3. Though many contrasting theories can be put forward, no one theroy can be particularly supported by the data. Thus this observation has to simply be regarded as experimental variation / error.

# Supplementary References

1 Iglewicz, B. & Hoaglin, D. C. *How to detect and handle outliers*. (ASQC Quality Press, 1993).

2 Sambrook, J. & Russell, D. W. Standard Ethanol Precipitation of DNA in Microcentrifuge Tubes. *Cold Spring Harbor Protocols* **2006**, pdb.prot4456, doi:10.1101/pdb.prot4456 (2006).
