# Supplementary material for: GC bias affects genomic and metagenomic reconstructions, underrepresenting GC-poor organisms
Source: Gigascience. 2020 Feb 13;9(2):giaa008. doi: 10.1093/gigascience/giaa008 (PMC7016772; doi:10.1093/gigascience/giaa008)
Supplement: giaa008_Supplemental_Files [file giaa008_supplemental_files.zip › Additional file 3.docx]

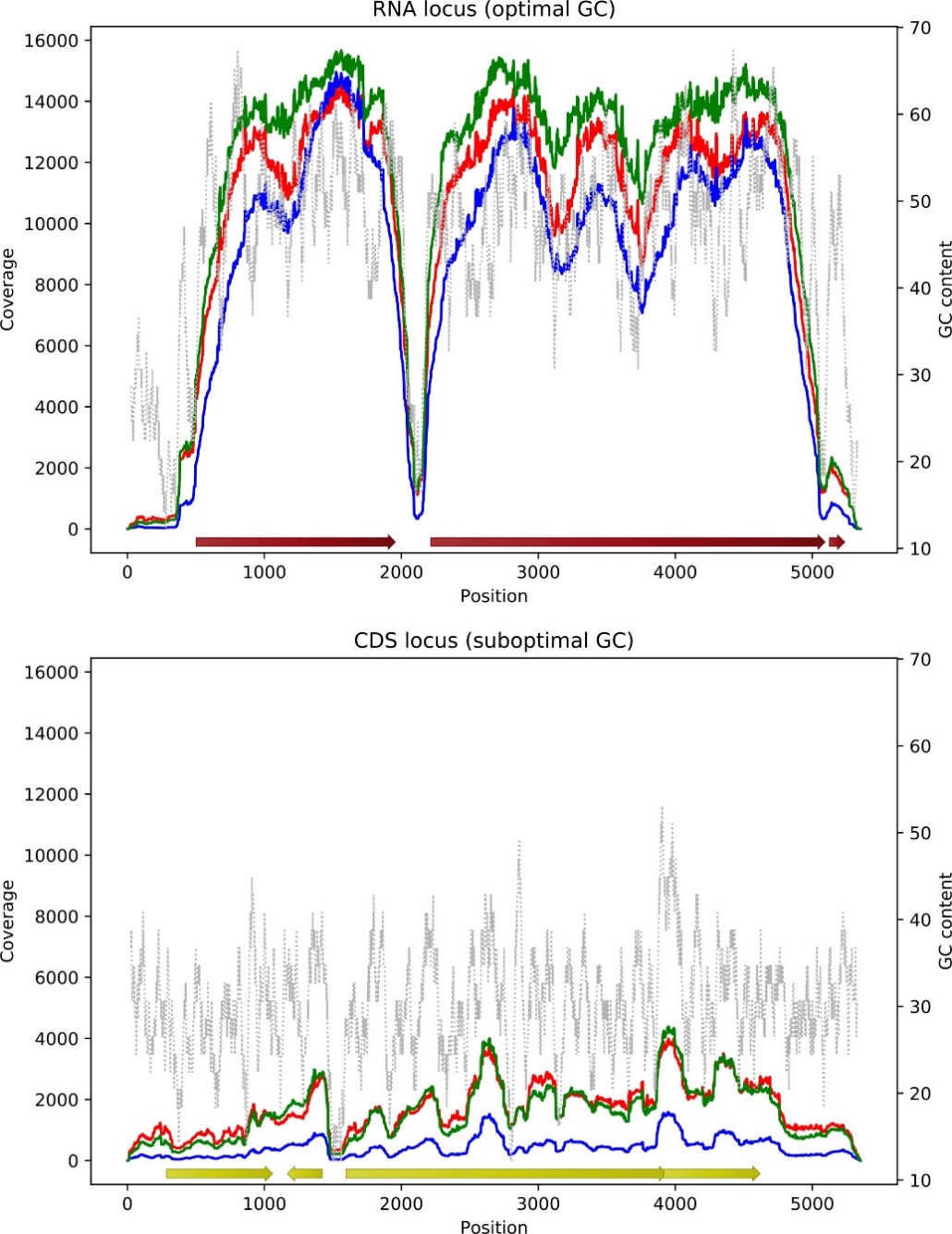
**Supplementary Figure 1** Coverage depths at every single nucleotide position and percent GC contents in 49 nt sliding windows. Percent GC content was calculated as the GC content of a nucleotide, with the 24 nucleotides downstream and the 24 nucleotides upstream (49 nucleotides in total). GC contents are not plotted for the first and last 24 nucleotides. Replicate 1: red line, Replicate 2: blue line, Replicate 3: green line, GC content: dotted grey line. The primary (coverage) and secondary (GC content) y-axes are scaled identically in both plots. The positions of the rRNA-encoding genes (red arrows) and protein coding genes (yellow arrows) are shown at the bottom of the respective plots.
