# Supplementary material for: GC bias affects genomic and metagenomic reconstructions, underrepresenting GC-poor organisms
Source: Gigascience. 2020 Feb 13;9(2):giaa008. doi: 10.1093/gigascience/giaa008 (PMC7016772; doi:10.1093/gigascience/giaa008)
Supplement: giaa008_Supplemental_Files [file giaa008_supplemental_files.zip › Additional file 4.docx]

**Supplementary Table 2** Numbers of reads mapped to two 5.3 kb equimolar PCR products from *Fusobacterium*.

|  | 30.2% GC mapped reads | 45.5% GC mapped reads | coverage ratio (45.5%/30.2%) |
| --- | --- | --- | --- |
| Replicate 1 | 49975 | 207027 | 4.14 |
| Replicate 2 | 13312 | 141459 | 10.63 |
| Replicate 3 | 42002 | 226509 | 5.39 |

Two ca. 5.3 kb equimolar PCR products with different GC contents from *Fusobacterium* were subjected to NexteraXT library preparation and MiSeq sequencing. The experiment had a paired design for each replicate. Either or both reads of a pair mapping was counted as one read mapped.
