# Supplementary material for: GC bias affects genomic and metagenomic reconstructions, underrepresenting GC-poor organisms
Source: Gigascience. 2020 Feb 13;9(2):giaa008. doi: 10.1093/gigascience/giaa008 (PMC7016772; doi:10.1093/gigascience/giaa008)
Supplement: giaa008_Supplemental_Files [file giaa008_supplemental_files.zip › Additional file 5.docx]

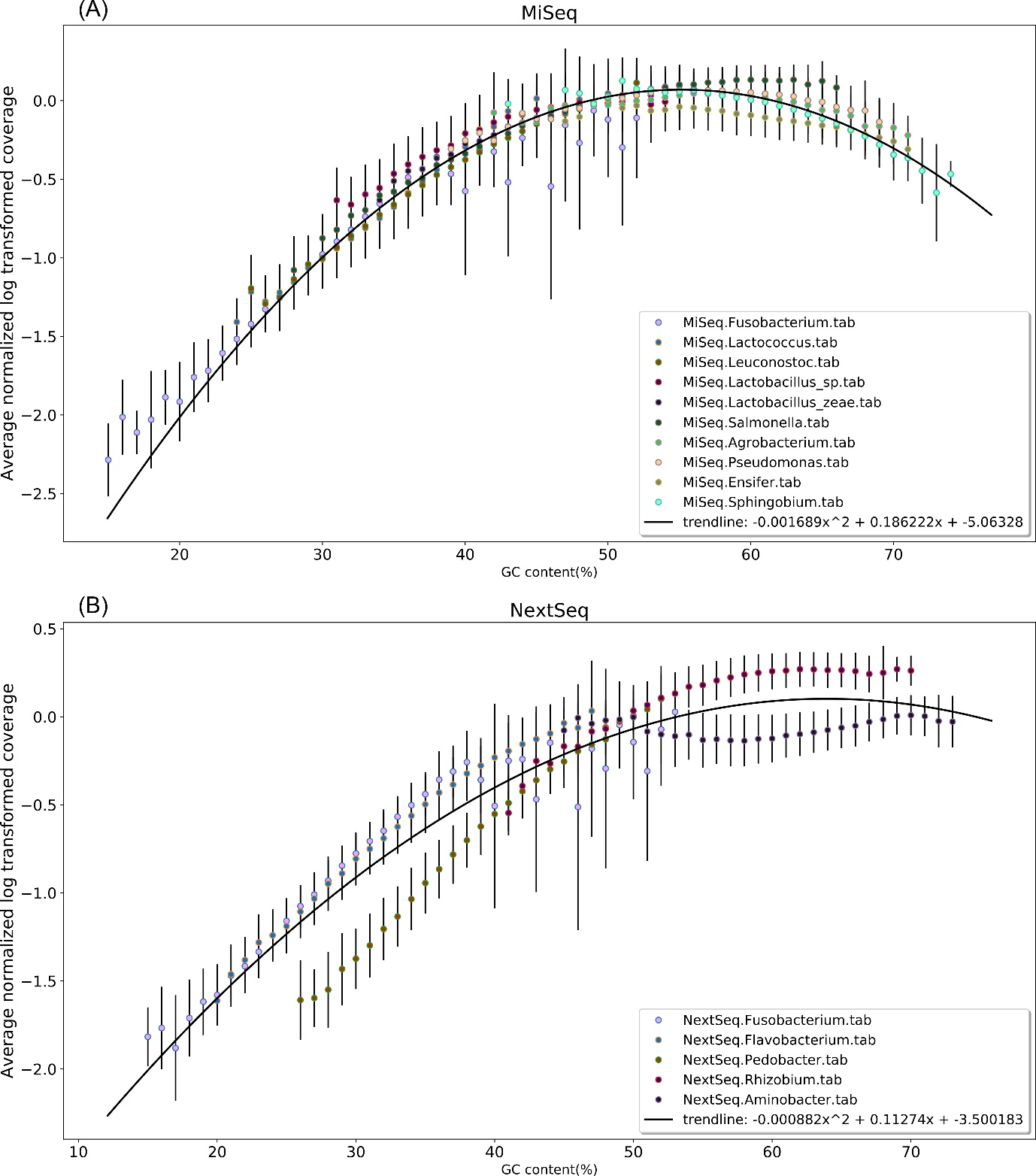


**Supplementary Figure 2** Plots illustrating the relationships between coverage and GC content. Normalized, log-transformed (base-10) coverage data from genome sequencing experiments of several organisms are shown for the MiSeq platform (A) and the NextSeq platform (B). The solid black lines represent the quadratic lines of best fit.
