# Supplementary figures and images for: GC bias affects genomic and metagenomic reconstructions, underrepresenting GC-poor organisms
Source: Gigascience. 2020 Feb 13;9(2):giaa008. doi: 10.1093/gigascience/giaa008 (PMC7016772; doi:10.1093/gigascience/giaa008)

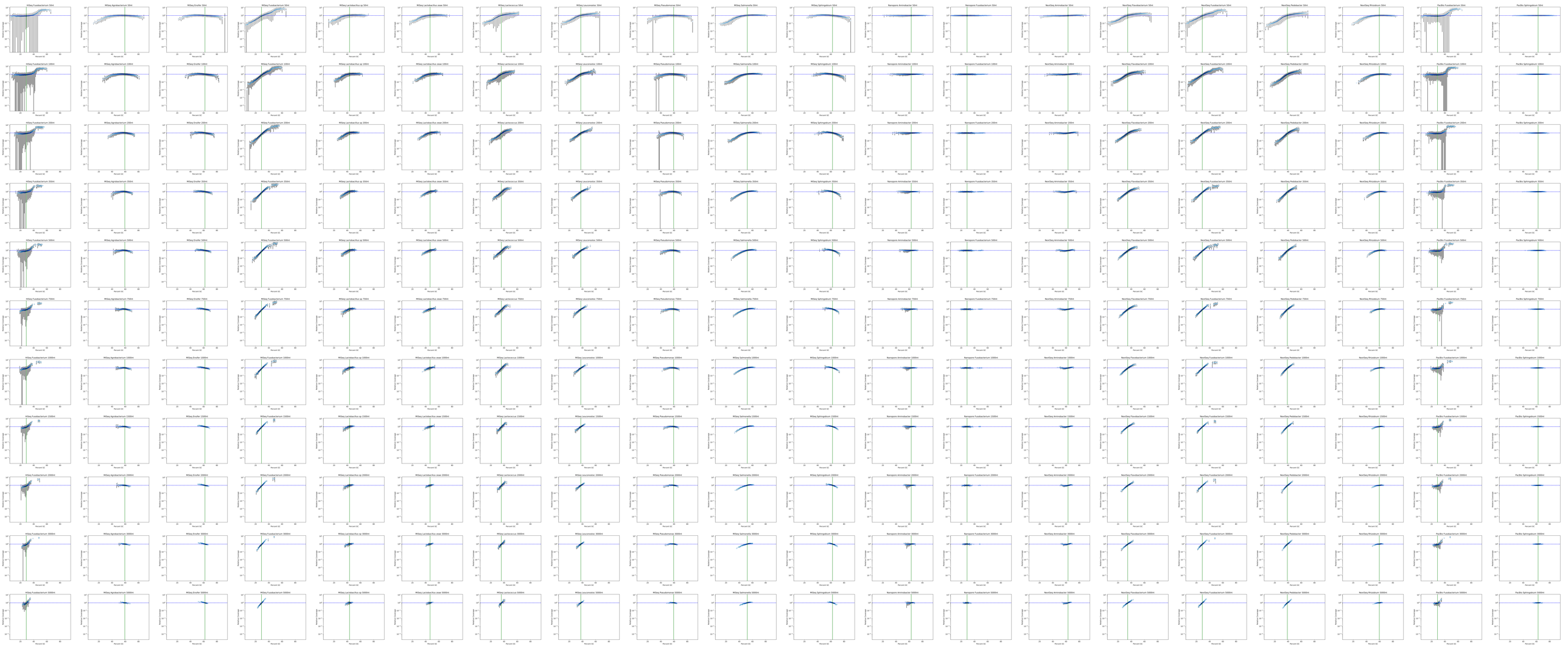

Supplement: giaa008_Supplemental_Files [file giaa008_supplemental_files.zip › Additional file 14.png]

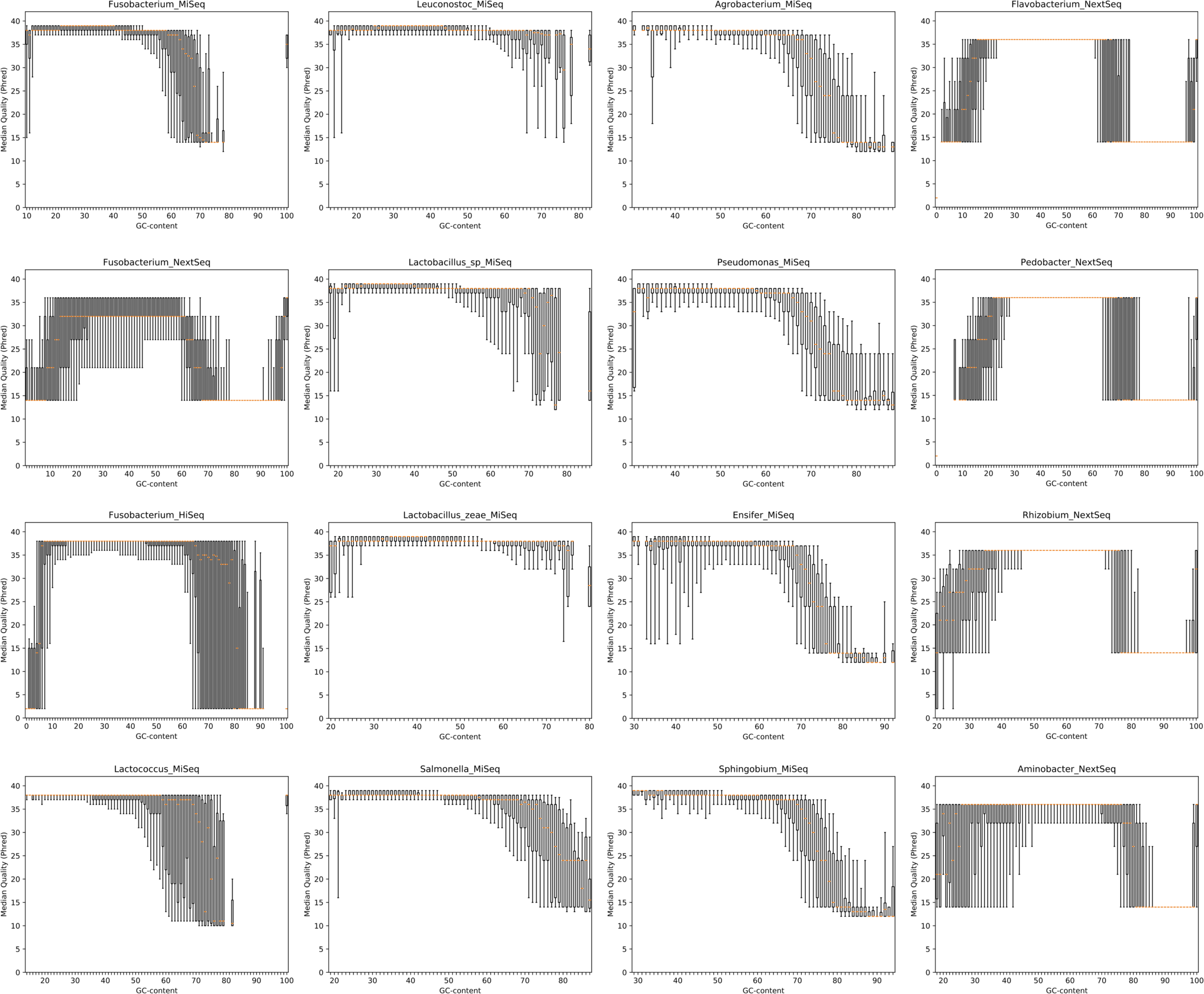

Supplement: giaa008_Supplemental_Files [file giaa008_supplemental_files.zip › Additional file 6.png]

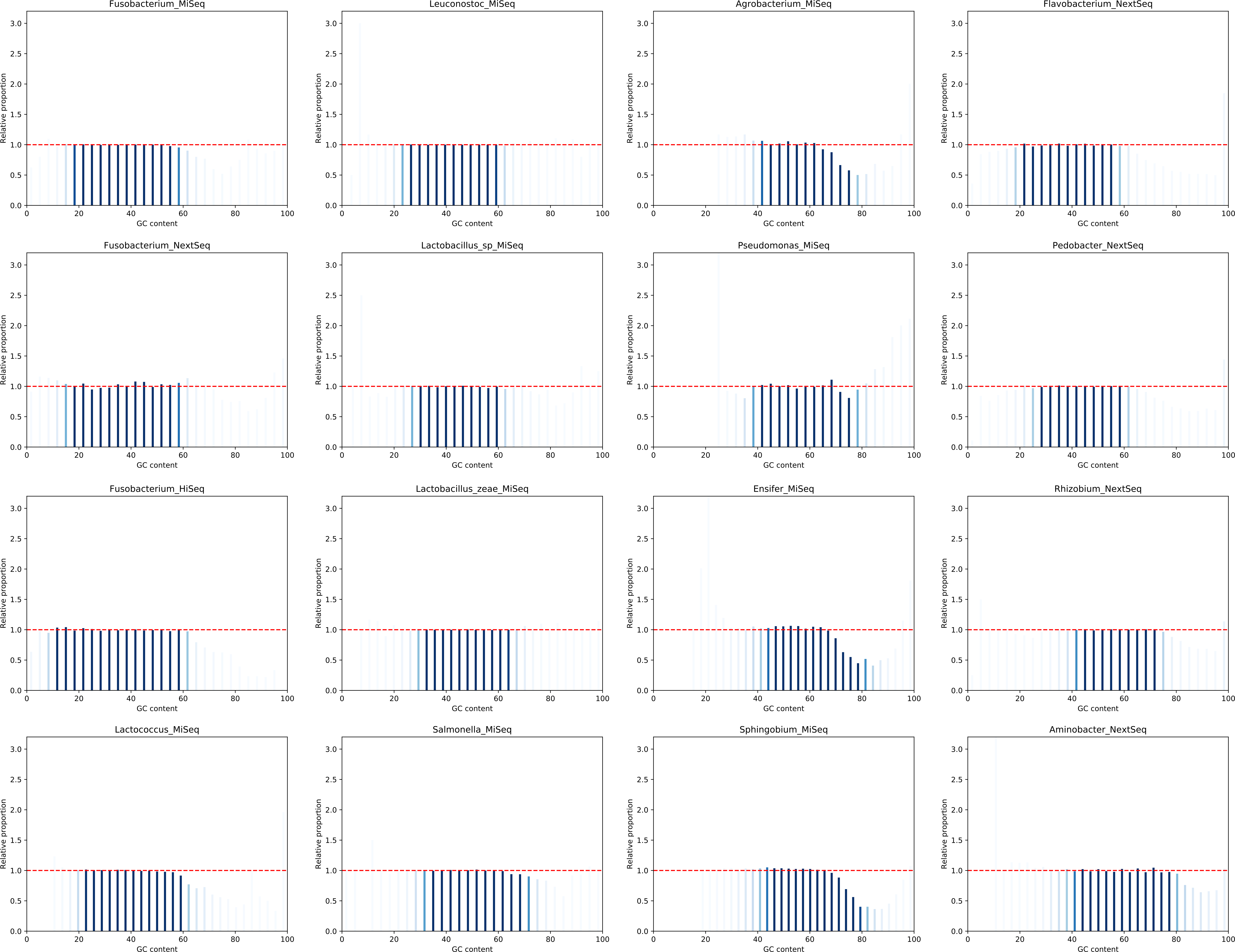

Supplement: giaa008_Supplemental_Files [file giaa008_supplemental_files.zip › Additional file 7.png]
