# Supplementary material for: GC bias affects genomic and metagenomic reconstructions, underrepresenting GC-poor organisms
Source: Gigascience. 2020 Feb 13;9(2):giaa008. doi: 10.1093/gigascience/giaa008 (PMC7016772; doi:10.1093/gigascience/giaa008)
Supplement: giaa008_Response_to_Reviewer_Comments_Original_Submission [file giaa008_response_to_reviewer_comments_original_submission.pdf]

# Author's Response To Reviewer Comments

Close

Reviewer #1
Browne et al present their results when studying GC-biases across several NGS platforms and for several microbial genomes.
While this is an important topic with applications/consequences in data analysis (e.g., assembly), several unclear, convoluted and confusing statements were found or many necessary information for validation/reproducibility were missing (cf. examples below):
Major issues:
- Methods:
- - "Coverage was assessed in 500 bp wide sliding windows, and the coverage was normalized by dividing by the average coverage of the 49% GC-genomic windows as all bacteria sequenced in this work have sufficient numbers of genomic windows with 49% GC content". Please provide references justifying this normalization method.
Response:
A reference to using a windowed approach similar to our approach is now inserted into the relevant methods section (lines 604 to 605). Also, further analyses justifying the choice of 500 nt as the window size was inserted into the same methods section (lines 613 to 619) and illustrated in a new supplementary figure (Additional file 14).

- - Why does the relative coverage decreases for high G+C content in half of the bacteria showed in Fig 2? please provide some explanations/insights.
Response:
We regret this oversight and agree that it is important to discuss this matter. The focus of this work was assessing the occurrence of GC bias in NGS datasets and our experiments were not designed to investigate the mechanisms responsible for introducing bias. Nonetheless, further analyses revealed that the likely cause for this is that the Illumina MiSeq sequencer yielded lower quality scores for high GC content reads. This resulted in quality filtering disproportionately filtering out high GC content reads. Thus we concluded that the source of the bias is largely due to an inability of the sequencer to call bases with high confidence (i.e. good Phred scores) in clusters with high GC content. This analysis and the results and conclusion were all added to the manuscript (lines 251 to 255, 273 to 276, 365 to 386, Additional files 6 + 7).

- - "The relatively small error-bars (standard deviation) seen in Fig. 2 indicate that relative coverage and local GC-content are tightly correlated." => I do not see how this statement is true. A small error-bars only indicates that the measurement method is itself precise, please fully explain what/why this correlation.
Response:
We are sorry for the need to clarify. Because the error bars represent the variability in the relative coverage of all the different 500 nt windows for each respective 1%-wide GC-bin, rather than a repeated measure of the same genomic region in different replicates, we disagree with the reviewer on this point. We have changed the text in order to make it clearer that the error bars represent the standard deviation of measurements of coverage among all 500 nt windows at each 1%-wide GC-bin (lines 242 to 244).

- - "Metagenome datasets were retrieved from several sources. Datasets ERR526087 (2 x 100bp) and SRR5035895 (2 x 300 bp) were retrieved with the fastq-dump utility of the SRA tookit V.2.9.0. The longest reads in these datasets were split in half and treated as read pairs, and shorter reads were discarded since the read pairs were concatenated without annotation of the concatenation point. " -> These datasets are Illumina Paired-end reads, hence why the need to split them and treat them as paired if they are paired already ? Also, all reads have same length in each dataset, hence how authors selected those that are the longest and those that are the shortest, if they all have same length...
Response:
In the SRA, reads may be stored with the pairs interleaved or concatenated. In the above-mentioned

SRA datasets, the read pairs were concatenated. When the reads are concatenated, there is no spacer nor filler sequence separating the reads. When reads are truncated in any way (e.g. when quality trimmed reads are uploaded to the SRA instead of raw reads) it is impossible to tell where the concatenated read should be split in order to recover the original R1 and R2 read pairs. Only in the case where neither of the reads in a pair were trimmed before concatenation is it possible to retrieve the original read pairs by splitting the paired read in half. For this reason, it is correct to keep only the full length reads and to then split them in half to retrieve the original pairs. This problem is described by Robert Edgar in his usearch v11 documentation for the fastq_sra_splitpairs command:
https://www.drive5.com/usearch/manual/cmd_fastq_sra_splitpairs.html
The manuscript was updated in order to make this problem clearer and to make it absolutely clear that single reads were not simply being split in two and treated as read pairs (lines 626 to 630).

- - Regarding the DNA extraction of the Fusabacterium sp. C1 isolates, how was it performed exactly (manual ? automated? kits used?...) ?
Response:
It is clearly stated in the relevant materials and methods section (Genome sequencing, assembly and annotation) that all DNA extractions were performed with the UltraClean Microbial DNA kit (MoBio) except for the DNA extracts for ddPCR and Nanopore sequencing, which were performed using the Genomic Mini AX Bacteria kit (A&A Biotechnology). Following the reviewer's comment, the word "experiment" was added after "ddPCR" in the relevant section of the text (line 555) as it could be misconstrued that the term "ddPCR library" was implied, which would be wrong and thus lead to confusion about DNA extraction methodologies.

- Results:
- - The poor quality of the figures provided, especially fig. 1, 2, is problematic and it does not permit the reader to quickly confirm/evaluate the explanations/claims that are made from them.
Response:
It is not clear in what way the reviewer means that the figures are of poor quality. Perhaps it is that they were in low-resolution in the PDF provided for review and the reviewer had a problem with the link in the pdf to access the high-resolution versions. We have now verified that these figures are of sufficient quality to be viewed clearly in the resolution intended for publication and we will accommodate the requests of the journal's copy editors in these matters should the need arise.

- - Authors claimed that their data were deposited under the Bioproject "PRJNA503577", yet the search engine in SRA/NBCI returns no result. Where is the data of this project?
Response:
This is indeed the correct BioProject number. The data is already uploaded to SRA, but will not be made publicly available until the date of publication. During the submission of this manuscript I didn't think to obtain a reviewer link to this data. I hereby apologize to the reviewers and editor for this oversight. The data under this BioProject number should be available for review at the following URL:
https://dataview.ncbi.nlm.nih.gov/object/PRJNA503577?reviewer=bajmo4nn0pv6gg3m0n28v9kbjt

- Other:
Authors focused their analysis almost all about the GC-content, yet the title refers to the AT-content. Authors should clarify/revise the title to reflect the content/results of their study.
Response:
The manuscript, including the title, was revised to address this issue and to make the terminology consistent. Terms referring to high AT or low AT or AT bias were replaced by suitable terms referring to GC.

Minor issues:
- Additional Table 1, I recommend authors to indicate the N50 for the pacbio and nanopore datasets, in addition to the minimum/median/maximum already provided.
Response:
It's a good suggestion. N50 values for pacbio and nanopore datasets have now been added to Additional Table 1.

- I believe the reader would be grateful if the authors can revise the many long paragraphs present in the manuscript into more concise ones.
Response:

Many changes are now made throughout this revised version to make it more readable.

Other General comments:
- Several grammatical English typo/mistakes were found (e.g., "well-establish" -> "well-established",
Response:
The correction was made exactly as suggested

"genomic and metagenomics data" -> "genomic and metagenomic data",
Response:
The correction was made exactly as suggested

"every more" -> "even more",
Response:
The intended meaning, obfuscated by the typo, was “ever more”. This has now been corrected.

"to increase understanding" -> "to increase the/our understanding" (?), etc.)
Response:
“to increase understanding” was changed to “to improve the general understanding”

and, often sentences are convoluted (for example, "PCR product sequencing depth investigation", this is not a correct English), please have the manuscript reviewed by a third-person skilled in English.
Response:
This is now changed to “Long range PCR product sequencing”. The manuscript has been reviewed by two native English speakers.

Reviewer #2
In this paper, Browne et al., attempt to systematically measure performances across various sequencing platforms using samples containing different level of GC content. While this a known issue (particularly for Illumina technologies) this is a useful analysis to quantify the potential impact on the accuracy of genomic and metagenomic reconstructions. Importantly, they have made all sequence data available at SRA and their analysis tools available via github allowing other labs to perform similar analyses, an important point given the suspected lab-specific biases. Overall, I believe the body of work is an important analysis highlighting significant technological biases whose impact is underappreciated. The following issues need to be addressed.

Major:
1)Did you try any other sliding window sizes and if so what did you observe? Why did you choose 500bp? The choice of window size may be impacted by the 'proximity to a region if balanced GC content' mentioned in line 353 in the discussion.
Response:
We did consider this point, but failed to discuss it in the text. A new supplementary file was added illustrating the same analyses using various different window sizes ranging from 50bp to 5000 bp. These are presented in a new supplementary figure (Additional file 14) and show that the conclusions are not affected by the choice of window sizes, although small window sizes showed more variability in the normalized coverages (error bars), while larger windows led to a reduction in the range of GC contents being represented in the data. Some details about these observations were also added to the relevant methods section (lines 613 to 619).

2)Did the authors examine reads with very high or low GC content for differences in base qualities relative to balanced GC content reads? Given QC software was utilized to trim/filter reads prior to alignment, it should be confirmed that high/low GC content reads were not being removed or trimmed extensively during QC prior to alignment.
Response:
The qualities of sequencing reads were investigated with respect to GC-content. Furthermore, the effects of quality filtering were investigated to see if quality filtering was impacting coverage in a manner related to GC content. It was concluded that the lowering of relative coverage above c.a. 65% GC content in certain MiSeq datasets is due to reads with high-GC content having lower quality and being disproportionately affected by quality filtering. However, we still maintain that the inability of a sequencer to produce base calls with a high-degree of certainty in high-GC regions is a subset of what we should refer to as GC bias. These effects were stated in the relevant analyses sections and discussed

in the discussion section and represented with two further supplementary figures (lines 252 to 256, 273 to 276, 365 to 386, Additional files 6 + 7). We thank the reviewer for making this interesting point because addressing it has added considerable value to this manuscript.

3)While the genomic analysis of the variable GC content in bacterial genomes illustrates a very clear and systematic contribution from GC content, the trend in the metagenomic analysis is less clear with five distinct profiles reported across the five data sets due to other cofounders. The authors make claims regarding the possibility of correcting for GC content in metagenomics (Line 403) however I am not sure this claim is supported by the analysis.
Response:
We perhaps stated this too generally. What we mean is that the GC bias within a metagenome dataset needs to be assessed following a metagenome assembly of that dataset in order to obtain parameters that could be used to correct abundance estimates. However, we did not explore the correction of GC bias in this work. We have now restated the relevant point to make it clear that we do not mean that the error profiles in our datasets here could somehow be used to correct GC biases in metagenome datasets in general (lines 448 to 452).

4)To verify the coverage spikes observed in Fig 1, the authors perform ddPCR and sequence two regions contain 30.2% and 45.5% GC content using an equimolar mixture. Overall, the 45.5% GC region mapped ~4X, ~11X, and 5X more reads than the 30.2% region. While the trend is clear, I would expect these numbers to be much closer however one replicate is overrepresented 3 times more than the other two replicates. Did you investigate if there is something substantially different about this replicate?
Response:
The authors have previously noted and discussed this difference. A lot of ideas have been put forward but none can be supported by our data. Therefore, we are up-front about the fact that there is a big variation in this experiment, but it can only be regarded as experimental (technical) variability. As the trends in coverage are similar among all replicates we assert that the data still supports the notion that the 45.5% GC regions receive much more coverage than the 30.2% GC region in our MiSeq workflow. We have now added a note to the relevant section of additional file 2 (the final paragraph of additional file 2) in order to discuss this point.

5)In the discussion (line 426), the authors point out their analysis is in some aspects, contradictory to several published works and indicate this is likely due to differences between labs which employ different library production protocols and HTS workflows. This is a critical finding of the analysis and needs to be stated more clearly throughout.
Response:
This is a good point as drawing attention to the major methodological differences between the different sequencing work flows is a good service to the reader who can now more easily ascertain which work flow led to which GC bias profile. This was addressed by adding a statement to the abstract (lines 58 to 59) that one of the key results was that library preparation and sequencing protocols affect the profile of GC bias. Furthermore, attention was drawn to the broad (and important) similarities and differences between methods producing data sets analysed in this work in the Data Description section (lines 158 to 164), and brief statements regarding the library production protocols were made while presenting the results (lines 230 to 231, 259 to 260, 277, 290 to 291 and 292).

6)This work looks at several different technologies and illustrates platform specific biases in their handling of different levels of GC content. With projects increasingly incorporating multiple sequencing technologies, it would be useful to discuss ideas for how best to combine the different platforms to minimize the impact of such biases.
Response:
This idea was mentioned in the discussion. However, an addition was made to make the meaning more obvious (lines 410 to 416).

Minor:
1)Central to many reported differences are issues in library production protocols. Given the apparent clustering of patterns in GC bias for different sequencing technologies, the authors need to more clearly define the protocols particularly with regard to similarities and differences.
Response:
The differences (and similarities) between the library production protocols are distilable from the relevant materials and methods section. However, we agree that this requires significant effort on a reader’s part to follow how the major differences between library production protocols may be related to

the GC-bias profiles presented in this work. In the Analyses section, there are now mentions about the major steps involved in each workflow which should make it easier for a reader to assess which protocol is associated with a particular GC-bias profile (lines 230 to 231, 259 to 260, 277, 290 to 291 and 292).

2)Throughout the manuscript, the authors jump from GC to AT content depending on context. It would be easier to follow if they consistently reported it with GC content listed first throughout.
Response:
The manuscript was revised to make terminology consistent. Terms referring to high AT or low AT or AT bias were replaced by terms referring to the relevant GC content.

3)Abstract typo: Metagenomic sequencing is a well-establish(ed) tool in the modern biosciences
Response:
The correction was made exactly as suggested

Close
