# Supplementary material for: GC bias affects genomic and metagenomic reconstructions, underrepresenting GC-poor organisms
Source: Gigascience. 2020 Feb 13;9(2):giaa008. doi: 10.1093/gigascience/giaa008 (PMC7016772; doi:10.1093/gigascience/giaa008)
Supplement: giaa008_Response_to_Reviewer_Comments_Revision_1 [file giaa008_response_to_reviewer_comments_revision_1.pdf]

Author's Response To Reviewer Comments

Close

An additional funding source was included in the Funding section.

The GigaDB dataset was cited in under 'Availability of supporting data and materials' and listed in the bibliography

Close
