# Supplementary material for: GC bias affects genomic and metagenomic reconstructions, underrepresenting GC-poor organisms
Source: Gigascience. 2020 Feb 13;9(2):giaa008. doi: 10.1093/gigascience/giaa008 (PMC7016772; doi:10.1093/gigascience/giaa008)
Supplement: giaa008_Reviewer_1_Report_Original_Submission — Rachid Ounit -- 8/18/2019 Reviewed [file giaa008_reviewer_1_report_original_submission.pdf]

Reviewer Report

Title: Substantial GC-bias impacts genomic and metagenomic reconstructions, significantly underrepresenting GC-poor organisms

Version: Original Submission **Date:** 8/18/2019

Reviewer name: Rachid Ounit

Reviewer Comments to Author:

Browne et al present their results when studying GC-biases across several NGS platforms and for several microbial genomes.
While this is an important topic with applications/consequences in data analysis (e.g., assembly), several unclear, convoluted and confusing statements were found or many necessary information for validation/reproducibility were missing (cf. examples below):
Major issues:
- Methods:
- - "Coverage was assessed in 500 bp wide sliding windows, and the coverage was normalized by dividing by the average coverage of the 49% GC-genomic windows as all bacteria sequenced in this work have sufficient numbers of genomic windows with 49% GC content". Please provide references justifying this normalization method.
- - Why does the relative coverage decreases for high G+C content in half of the bacteria showed in Fig 2? please provide some explanations/insights.
- - "The relatively small error-bars (standard deviation) seen in Fig. 2 indicate that relative coverage and local GC-content are tightly correlated." => I do not see how this statement is true. A small error-bars only indicates that the measurement method is itself precise, please fully explain what/why this correlation.
- - "Metagenome datasets were retrieved from several sources. Datasets ERR526087 (2 x 100bp) and SRR5035895 (2 x 300 bp) were retrieved with the fastq-dump utility of the SRA tookit V.2.9.0. The longest reads in these datasets were split in half and treated as read pairs, and shorter reads were discarded since the read pairs were concatenated
without annotation of the concatenation point. " -> These datasets are Illumina Paired-end reads, hence why the need to split them and treat them as paired if they are paired already ? Also, all reads have same length in each dataset, hence how authors selected those that are the longest and those that are the shortest, if they all have same length...
- - Regarding the DNA extraction of the Fusabacterium sp. C1 isolates, how was it performed exactly (manual ? automated? kits used?...) ?
- Results:
- - The poor quality of the figures provided, especially fig. 1, 2, is problematic and it does not permit the reader to quickly confirm/evaluate the explanations/claims that are made from them.
- - Authors claimed that their data were deposited under the Bioproject "PRJNA503577", yet the search engine in SRA/NBCI returns no result. Where is the data of this project?

- Other:
Authors focused their analysis almost all about the GC-content, yet the title refers to the AT-content. Authors should clarify/revise the title to reflect the content/results of their study.
Minor issues:
- Additional Table 1, I recommend authors to indicate the N50 for the pacbio and nanopore datasets, in addition to the minimum/median/maximum already provided.
- I believe the reader would be grateful if the authors can revise the many long paragraphs present in the manuscript into more concise ones.
Other General comments:
- Several grammatical English typo/mistakes were found (e.g., "well-establish" -> "well-established",
"genomic and metagenomics data" -> "genomic and metagenomic data",
"every more" -> "even more",
"to increase understanding" -> "to increase the/our understanding" (?), etc.)
and, often sentences are convoluted (for example, "PCR product sequencing depth investigation", this is not a correct English), please have the manuscript reviewed by a third-person skilled in English.

Methods

Are the methods appropriate to the aims of the study, are they well described, and are necessary controls included? Choose an item.

Conclusions

Are the conclusions adequately supported by the data shown? Choose an item.

Reporting Standards

Does the manuscript adhere to the journal's guidelines on [minimum standards of reporting?] Choose an item.

Choose an item.

Statistics

Are you able to assess all statistics in the manuscript, including the appropriateness of statistical tests used? Choose an item.

Quality of Written English

Please indicate the quality of language in the manuscript: Choose an item.

Declaration of Competing Interests

Please complete a declaration of competing interests, considering the following questions:

- Have you in the past five years received reimbursements, fees, funding, or salary from an organisation that may in any way gain or lose financially from the publication of this manuscript, either now or in the future?
- Do you hold any stocks or shares in an organisation that may in any way gain or lose financially from the publication of this manuscript, either now or in the future?
- Do you hold or are you currently applying for any patents relating to the content of the manuscript?
- Have you received reimbursements, fees, funding, or salary from an organization that holds or has applied for patents relating to the content of the manuscript?
- Do you have any other financial competing interests?
- Do you have any non-financial competing interests in relation to this paper?

If you can answer no to all of the above, write 'I declare that I have no competing interests' below. If your reply is yes to any, please give details below.

I declare that I have no competing interests

I agree to the open peer review policy of the journal. I understand that my name will be included on my report to the authors and, if the manuscript is accepted for publication, my named report including any attachments I upload will be posted on the website along with the authors' responses. I agree for my report to be made available under an Open Access Creative Commons CC-BY license (http://creativecommons.org/licenses/by/4.0/). I understand that any comments which I do not wish to be included in my named report can be included as confidential comments to the editors, which will not be published.

Choose an item.

To further support our reviewers, we have joined with Publons, where you can gain additional credit to further highlight your hard work (see: https://publons.com/journal/530/gigascience). On publication of this paper, your review will be automatically added to Publons, you can then choose whether or not to claim your Publons credit. I understand this statement.

Yes Choose an item.
