# Supplementary material for: GC bias affects genomic and metagenomic reconstructions, underrepresenting GC-poor organisms
Source: Gigascience. 2020 Feb 13;9(2):giaa008. doi: 10.1093/gigascience/giaa008 (PMC7016772; doi:10.1093/gigascience/giaa008)
Supplement: giaa008_Reviewer_2_Report_Original_Submission — Matt Field, PhD -- 8/29/2019 Reviewed [file giaa008_reviewer_2_report_original_submission.pdf]

Reviewer Report

Title: Substantial GC-bias impacts genomic and metagenomic reconstructions, significantly underrepresenting GC-poor organisms

Version: Original Submission **Date:** 8/29/2019

Reviewer name: Matt Field, PhD

Reviewer Comments to Author:

In this paper, Browne et al., attempt to systematically measure performances across various sequencing platforms using samples containing different level of GC content. While this a known issue (particularly for Illumina technologies) this is a useful analysis to quantify the potential impact on the accuracy of genomic and metagenomic reconstructions. Importantly, they have made all sequence data available at SRA and their analysis tools available via github allowing other labs to perform similar analyses, an important point given the suspected lab-specific biases. Overall, I believe the body of work is an important analysis highlighting significant technological biases whose impact is underappreciated. The following issues need to be addressed.

Major:

1) Did you try any other sliding window sizes and if so what did you observe? Why did you choose 500bp? The choice of window size may be impacted by the 'proximity to a region if balanced GC content' mentioned in line 353 in the discussion.

2) Did the authors examine reads with very high or low GC content for differences in base qualities relative to balanced GC content reads? Given QC software was utilized to trim/filter reads prior to alignment, it should be confirmed that high/low GC content reads were not being removed or trimmed extensively during QC prior to alignment.

3) While the genomic analysis of the variable GC content in bacterial genomes illustrates a very clear and systematic contribution from GC content, the trend in the metagenomic analysis is less clear with five distinct profiles reported across the five data sets due to other cofounders. The authors make claims regarding the possibility of correcting for GC content in metagenomics (Line 403) however I am not sure this claim is supported by the analysis.

4) To verify the coverage spikes observed in Fig 1, the authors perform ddPCR and sequence two regions contain 30.2% and 45.5% GC content using an equimolar mixture. Overall, the 45.5% GC region mapped ~4X, ~11X, and 5X more reads than the 30.2% region. While the trend is clear, I would expect these numbers to be much closer however one replicate is overrepresented 3 times more than the other two replicates. Did you investigate if there is something substantially different about this replicate?

5) In the discussion (line 426), the authors point out their analysis is in some aspects, contradictory to several published works and indicate this is likely due to differences between labs which employ different library production protocols and HTS workflows. This is a critical finding of the analysis and needs to be stated more clearly throughout.

6) This work looks at several different technologies and illustrates platform specific biases in their

handling of different levels of GC content. With projects increasingly incorporating multiple sequencing technologies, it would be useful to discuss ideas for how best to combine the different platforms to minimize the impact of such biases.
Minor:
1) Central to many reported differences are issues in library production protocols. Given the apparent clustering of patterns in GC bias for different sequencing technologies, the authors need to more clearly define the protocols particularly with regard to similarities and differences.
2) Throughout the manuscript, the authors jump from GC to AT content depending on context. It would be easier to follow if they consistently reported it with GC content listed first throughout.
3) Abstract typo: Metagenomic sequencing is a well-establish(ed) tool in the modern biosciences

Methods

Are the methods appropriate to the aims of the study, are they well described, and are necessary controls included? Choose an item.

Conclusions

Are the conclusions adequately supported by the data shown? Choose an item.

Reporting Standards

Does the manuscript adhere to the journal's guidelines on minimum standards of reporting? Choose an item.

Choose an item.

Statistics

Are you able to assess all statistics in the manuscript, including the appropriateness of statistical tests used? Choose an item.

Quality of Written English

Please indicate the quality of language in the manuscript: Choose an item.

Declaration of Competing Interests

Please complete a declaration of competing interests, considering the following questions:

- Have you in the past five years received reimbursements, fees, funding, or salary from an organisation that may in any way gain or lose financially from the publication of this manuscript, either now or in the future?

- Do you hold any stocks or shares in an organisation that may in any way gain or lose financially from the publication of this manuscript, either now or in the future?
- Do you hold or are you currently applying for any patents relating to the content of the manuscript?
- Have you received reimbursements, fees, funding, or salary from an organization that holds or has applied for patents relating to the content of the manuscript?
- Do you have any other financial competing interests?
- Do you have any non-financial competing interests in relation to this paper?

If you can answer no to all of the above, write 'I declare that I have no competing interests' below. If your reply is yes to any, please give details below.

I declare that I have no competing interests

I agree to the open peer review policy of the journal. I understand that my name will be included on my report to the authors and, if the manuscript is accepted for publication, my named report including any attachments I upload will be posted on the website along with the authors' responses. I agree for my report to be made available under an Open Access Creative Commons CC-BY license (http://creativecommons.org/licenses/by/4.0/). I understand that any comments which I do not wish to be included in my named report can be included as confidential comments to the editors, which will not be published.

Choose an item.

To further support our reviewers, we have joined with Publons, where you can gain additional credit to further highlight your hard work (see: https://publons.com/journal/530/gigascience). On publication of this paper, your review will be automatically added to Publons, you can then choose whether or not to claim your Publons credit. I understand this statement.

Yes Choose an item.
